# Effect of colonisation with *Neisseria lactamica* on cross-reactive anti-meningococcal B cell responses: A randomised, controlled, human infection trial

**DOI:** 10.1016/S2666-5247(22)00283-X

**Published:** 2022-12-01

**Authors:** Adam P. Dale, Anastasia A. Theodosiou, Diane F. Gbesemete, Jonathan M. Guy, Eleanor F. Jones, Alison R. Hill, Muktar M. Ibrahim, Hans de Graaf, Muhammad Ahmed, Saul N. Faust, Andrew R. Gorringe, Marta E. Polak, Jay R. Laver, Robert C. Read

**Affiliations:** 1 Faculty of Medicine and Institute for Life Sciences, University of Southampton, Southampton, SO17 1BJ, UK.; 2 NIHR Southampton Biomedical Research Centre and NIHR Southampton Clinical Research Facility, University Hospital Southampton NHS Foundation Trust, Southampton, SO16 6YD, UK.; 3 UK Health Security Agency, Porton Down, Salisbury, SP4 0JG, UK.

## Abstract

**Background:**

Pharyngeal colonisation by the harmless commensal *Neisseria lactamica* (Nlac) inhibits *Neisseria meningitidis* (Nmen) colonisation and has an inverse epidemiological association with meningococcal disease. The mechanisms underpinning this relationship remain unexplained, but could be due to induction of cross-reactive immunity. In this study, we evaluated whether colonisation with Nlac induces Nlac-specific B cell responses cross-reactive with Nmen.

**Methods:**

In a randomised, placebo-controlled, human infection experiment at University Hospital Southampton Clinical Research Facility, UK, healthy adults, aged 18-45 years, were randomised 2:1 to receive intra-nasal inoculation with either 10^5^ colony-forming units of Nlac in 1 ml phosphate buffered saline (PBS), or 1 ml PBS alone. Participants, and researchers performing participant sampling and immunological assays, were all blinded to allocation. The primary endpoint was a comparison of circulating Nlac-specific plasma cell (B_PLAS_) and memory B cell (B_MEM_) frequencies post Nlac inoculation (day 7-28), as compared to baseline (day 0), measured using Enzyme-Linked ImmunoSpot (ELISpot) assays. The secondary endpoint was to measure the frequency of Nmen-specific B_PLAS_ and B_MEM_. The trial is registered with ClinicalTrials.gov (NCT03633474) and is now closed.

**Findings:**

*n* = 31/50 of participants assessed for eligibility between Sep 5, 2018, and Mar 3, 2019, were randomly assigned (*n*=20 Nlac, *n*=11 PBS). Amongst Nlac-colonised participants (*n*=17), median baseline compared to peak post-colonisation Nlac-specific B_PLAS_ frequencies (per 10^5^ Peripheral Blood Mononuclear Cells) were 0 (IQR 0.0-0.0) versus 5 (1.5-10.5) for IgA-secreting B_PLAS_ *(P* <0.0001), and 0 (0.0-0.0) versus 3 (1.5-9.5) for IgG-secreting B_PLAS_ (*P* <0.0001). Median Nlac-specific IgG B_MEM_ frequencies (% of total IgG B_MEM_) increased from 0.0024% (0.0000-0.0097) at baseline to 0.0384% (0.0275-0.0649) at day 28 (*P* <0.0001). The frequency of Nmen-specific IgA- and IgG-secreting B_PLAS_ and IgG B_MEM_ also increased significantly amongst Nlac-colonised participants. Nlac- and Nmen-specific B_PLAS_ and B_MEM_ were unchanged amongst controls. Upper respiratory tract symptoms were reported amongst *n*=10/20 Nlac-inoculated and *n*=6/11 PBS-inoculated participants (*P* >0.99). There were 3 additional adverse events and no serious adverse events.

**Interpretation:**

Natural immunity to Nmen following Nlac colonisation may be due to cross-reactive adaptive responses. Exploitation of this microbial mechanism with a genetically modified live vector could protect against Nmen colonisation and disease.

## Introduction

The human oro-nasal pharynx is colonised by a wide range of harmless commensals which provide natural immunity against occasional pathobionts. The mechanism in many cases is undetermined but could be harnessed for future vaccine strategies. Pharyngeal colonisation with the pathogen *Neisseria meningitidis* (Nmen) occurs in approximately ten percent of the population and is prerequisite for invasive meningococcal disease.^1^ While glycoconjugate meningococcal vaccines have dramatically reduced invasive meningococcal disease, largely due to induction of herd protection through population-level reduction of Nmen colonisation,^2,3^ protein-based serogroup B vaccines (4CMenB/MenB-FHbp) do not reduce serogroup B Nmen colonisation and are therefore unlikely to provide herd protection.^4–6^

*Neisseria lactamica* (Nlac) is non-capsulate and lacks pathogenic potential, mainly colonising the upper respiratory tract (URT) of children. Age-specific rates of Nmen colonisation and disease correlate inversely with Nlac colonisation prevalence,^7,8^ and experimental colonisation with Nlac protects against Nmen colonisation for at least 26 weeks.^9^ Mathematical modelling predicts that the protective effect of Nlac on Nmen persists for a number of years and colonisation with Nlac results in generation of anti-Nmen opsonophagocytic activity (OPA) in serum, but not anti-Nmen serum bactericidal activity (SBA).^10,11^ Furthermore, vaccination of mice with Nlac outer-membrane vesicles (OMV) protects against fatal meningococcal infection through a SBA-independent mechanism.^12^ These data support the hypothesis that generation of cross-reactive adaptive immune responses, independent of the formation of Nmen-specific SBA, may be implicated in natural protection afforded by Nlac colonisation on Nmen colonisation and disease.

Testing this hypothesis, we performed a randomised, placebo-controlled human infection model experiment (CHIME) with the primary aim of determining whether Nlac colonisation induced Nlac-specific plasma cell (B_PLAS_) and memory B cell (B_MEM_) responses in blood. The secondary aim was to establish whether Nlac-specific responses induced by Nlac colonisation were cross-reactive with Nmen. Generation of adaptive immune responses is likely timedependent, so in a second Nlac CHIME we investigated whether seroconversion was dependent on Nlac colonisation duration.

## Methods

### Study design and participants

In the first Nlac CHIME (hereafter, Study A), healthy adult participants, aged 18-45 years, were randomised 2:1 in permuted blocks of six to receive intra-nasal inoculation with intervention (10^5^ CFU Nlac in 1ml PBS [pH 7.4] [Severn Biotech]) or visually-identical control (1ml PBS alone), as previously outlined.^9^ A 2:1 ratio was chosen as inoculation with 10^4^-10^5^ CFU previously resulted in ~40-50 % colonisation.^9,11^ After inoculation, follow-up visits were performed at 7-, 14- and 28-days to determine Nlac/Nmen colonisation and immune responses (Supplementary Table 1). Participants who developed natural Nmen colonisation were excluded from immunological analyses to prevent confounding.

In the second Nlac CHIME (hereafter, Study B), participants were inoculated intra-nasally with 10^5^ CFU Nlac in 1ml PBS and then assigned to receive oral ciprofloxacin (500mg) at either 4 days or 14 days following inoculation. Samples were taken post-inoculation to determine Nlac/Nmen colonisation, and to assess IgG titres (Supplementary Table 2).

Both CHIMEs were performed at University Hospital Southampton Clinical Research Facility, UK. Inclusion and exclusion criteria are listed in Supplementary Table 3. Clinical study protocols are available at: https://doi.org/10.5258/SOTON/P1072/ https://doi.org/10.5258/SOTON/P1073. Study A was sponsored by the University of Southampton, UK (ERGO 32061), and reviewed by the South Central Oxford C Research Ethics Committee (REC), UK (18/SC/0311). Study B was sponsored by University Hospital Southampton NHS Foundation Trust, UK (RHM MED1354), and reviewed by the Hampshire A REC, UK (16/SC/0425). Both CHIMEs were overseen by independent safety committees and written informed consent was obtained from all participants prior to enrolment.

### Randomisation and masking

To maintain allocation concealment in Study A, a computer-generated allocation sequence was uploaded to online randomisation software (sealed envelope™) prior to study start, by a researcher not otherwise involved in the study. Assignment was subsequently performed on the day of inoculation using sealed envelope™ by a single study clinician who was also responsible for enrolment, preparation and instillation of inocula, and microbiological culture of relevant specimens. Participants, and researchers performing participant sampling and immunological assays, were all blinded to allocation. In Study B, neither participants nor researchers performing sampling were blinded to allocation. However, researchers performing Nlac- and Nmen-specific IgG Enzyme-Linked Immunosorbent Assays (ELISA) were blinded to allocation.

### Procedures

#### Preparation of inocula

Nlac inocula were prepared from stock vials (Y92-1009, sequence type 3493, clonal complex 613, in Frantz medium) produced in the Good Manufacturing Practice facilities at UK Health Security Agency (formerly, Public Health England).^13^ Control was visually-identical PBS. Inoculum purity and dose were determined by culture on Columbia Blood Agar (CBA) and gonococcal (GC) selective agar supplemented with 10 % (v/v) lysed horse blood, VCAT selective supplement (1 %, v/v), vitox supplement (2 %, v/v), glucose (0.4 %, w/v),amphotericin B (1 mg l^-1^) and 5-bromo-4-chloro-3-indolyl-B-D galactopyranoside (X-gal) (40 mg l^-1^), (Southern Group Laboratories) (GC-X-gal).

#### Culture and identification of *Neisseria* species

Nlac/Nmen colonisation was assessed by culture of oropharyngeal swabs (TS/5-17 Probact swab, Sterilin, with Amies transport medium) and nasal wash on GC-X-gal plates, as described previously.^13^ For the nasal wash, 10 ml of sterile saline (0·9 %, BLUE DOT) was instilled intra-nasally via each nostril in turn and left in situ for 60 seconds. If <5 ml nasal wash was returned, the procedure was repeated once more. Nasal wash was centrifuged at 5000 *g* for 10 minutes and the pellet resuspended in 300 μl PBS. Two-hundred and fifty microlitres and 25 μl of the nasal wash suspensions were spread over GC-X-gal plates prior to incubation. Nlac/Nmen growth was confirmed using biochemical methods.^9^ Nasal wash Nlac colonisation density was calculated as CFU ml^-1^. Blood was collected into 10 ml EDTA blood collection tubes (Vacutainer®, BD).

#### Symptom reporting

At each visit, participants were asked to report URT symptoms, other adverse events, and antibiotic exposure.

#### Isolation of deoxycholate-extracted OMV (dOMV) and PBMC

Nlac and Nmen dOMV were derived as described previously.^14^ Blood was centrifuged at 700 x *g* for 10 minutes and the plasma frozen at -80 °C. PBMC were isolated using density gradient centrifugation and used immediately or cryopreserved in liquid nitrogen.

#### Polyclonal stimulation of PBMC

PBMC were thawed and polyclonally stimulated as described previously with 3 μg ml^-1^ human phosphorothioate-modified oligodeoxynucleotide containing CpG motifs (ODN2006: 5’-TCG TCG TTT TGT CGT TTT GTC GTT-3’) (InvivoGen), 10 ng ml^-1^ IL-2 (R&D Systems), and 10 ng ml^-1^ IL-10 (Pharmingen, BD) for 5 days at 37 °C, 5% CO2, in AIM/V +albumax medium (Gibco, Invitrogen) supplemented with 10 % FCS and 50 μM β-mercaptoethanol (hereafter, AIM/V+).^15^

#### ELISpot assays

ELISpot assays were performed as described previously.^15^ Briefly, for B_PLAS_ ELISpot assays, ELISpot plates (Multiscreen HTS plate, Merck Millipore) were coated with 100 μl PBS containing: keyhole limpet haemocyanin (KLH) (naïve antigen control) (10 μg ml^-1^, Sigma-Aldrich), TT (2·5 level of flocculation units ml^-1^, National Institute for Biological Standards and Control − NIBSC), Nlac Y92-1009-dOMV (hereafter, Nlac-dOMV) (10 μg ml^-**1**^), Nmen H44/76-dOMV (hereafter, Nmen-dOMV) (10 μg ml^-**1**^), rat anti-human IgG mAb (clone M1310G05, IgG2a, k, BioLegend) (hereafter, anti-IgG mAb) (10 μg ml^-**1**^) or mouse anti-human IgA mAb (clone HP6123, IgG1, Biolegend) (hereafter, anti-IgA mAb) (10 μg ml^-1^). For B_MEM_ ELISpot assays, wells were coated with 100 μl PBS containing: KLH (10 μg ml^-1^), Influenza antigen reagent 09/174, H1N1 [NIBSC] (0·5 μg ml^-1^) (hereafter, Flu HA), Nlac-dOMV (10 μg ml^-**1**^), Nmen-dOMV (10 μg ml^-**1**^) or anti-IgG mAb (10 μg ml^-**1**^).

For B_PLAS_ ELISpot assays, 2 x 10^5^ freshly-isolated PBMC were seeded in duplicate in 200 μl AIM/V. For B_MEM_ ELISpot assays, polyclonally-stimulated PBMC were harvested, washed, and seeded in triplicate in 200 μl AIM/V at 1 x 10^6^ and 4 x 10^5^ cells well^-1^ (Nlac-dOMV-, Nmen-dOMV- and KLH-coated wells), 5 x 10^4^ cells well^-1^ (Flu HA- and KLH-coated wells), and 5 x 10^2^ cells well^-1^ (anti-IgG mAb- and KLH-coated wells).

Following incubation, wells were washed and incubated with secondary antibodies (ALK-P-conjugated goat anti-human IgA polyclonal antibody [pAb] [1:2500, Sigma-Aldrich] [hereafter, anti-IgA pAb] [B_PLAS_ ELISpot assay only] or ALK-P-conjugated goat-anti-human IgG pAb [1:10,000, Sigma-Aldrich] [hereafter, anti-IgG pAb] [B_PLAS_ and B_MEM_ ELISpot assays]) prior to development with 5-bromo-4-chloro-3-indolyl phosphate (BCIP) (Sigma-Aldrich). Wells were then washed, dried and imaged (AID® plate reader).

IgA and IgG spot-forming units (SFU) were enumerated using AID® ELISpot software, version 3·5, with standardised settings. For the B_PLAS_ ELISpot assay, mean B_PLAS_ frequencies were expressed as IgA or IgG SFU per 2 x 10^5^ PBMC, following subtraction of the mean SFU count in KLH-coated wells, assuming 1 SFU = 1 B_PLAS_. For the B_MEM_ ELISpot assay, antigen-specific IgG SFU were derived for Nlac-dOMV- and Nmen-dOMV-coated wells from the lowest input cell number where a mean of 1-20 IgG SFU were counted after subtraction of KLH background. Where membrane saturation occurred at 4 x 10^5^ well^-1^, experiments were repeated with 2 x 10^5^ and 5 x 10^4^ input cells well^-1^ to derive an accurate IgG SFU count. Antigen-specific IgG SFU were expressed as a percentage of total IgG SFU, as described previously.^16^ B_MEM_ ELISpot assay data were included in the B_MEM_ analysis if PBMC polyclonal stimulation was successful (IgG SFU in anti-IgG mAb-coated wells and ≥1 IgG SFU [mean] in Flu HA-coated wells, following subtraction of KLH background).

#### IgG ELISA

IgG ELISAs were performed as described previously.^15^ Briefly, plasma (Study A) or serum (Study B) derived from participants and a single-donor positive control were serially-diluted and loaded in duplicate into 96-well EIA/RIA plates (Corning Costar) pre-coated with the following antigens in 50 μl carbonate coating buffer (pH 9·6): (i) Nlac-dOMV (20 μg ml^-**1**^), (ii) Nmen-dOMV (20 μg ml^-**1**^), and (iii) BSA (20 μg ml^-**1**^). IgG was detected using biotinylated anti-IgG mAb (M1310G05) (BioLegend) with subsequent incubation with streptavidin horseradish peroxidase (HRP) in PBS/FCS (Abcam) prior to addition of the chromogenic substrate, o-phenylenediamine dihydrochloride (OPD) (Thermo Fischer Scientific). Colour change was quantified on a VersaMax plate reader (Molecular Diagnostics) measuring at OD490nm. A four-parameter logistic log curve was fitted to titrations of the reference plasma or serum for each plate. Participant sample IgG titres were calculated by interpolation from the reference dose-response curve (interpolated titre multiplied by dilution factor). A mean of the acceptable values from all dilutions was then taken as the final value for each test sample.

#### Outcomes

For Study A, the primary endpoint was to assess the change in frequency of Nlac-specific B_PLAS_ and B_MEM_ in blood amongst Nlac-colonised versus PBS-inoculated participants, comparing baseline (day 0) with post-inoculation timepoints (day 7-28). The secondary endpoint was to assess the change in frequency of Nmen-specific B_PLAS_ and B_MEM_. For Study B, the primary endpoint was to assess the rise in anti-Nlac IgG titre, comparing baseline with 14-days and 28-days post-Nlac eradication, amongst participants colonised with Nlac for 4 or 14 days.

#### Statistical analysis

For Study A, SDs associated with serological responses to Nlac colonisation from a previous Nlac CHIME were utilised to inform the sample size calculation.^11^ This gave SDs on a log_10_ scale of 0·26 for serum IgG. Using the SD of 0·26, it was calculated that a fourfold rise in Nlac-specific IgG titre would be confirmed with ten Nlac colonised participants with 90 % power using analysis of variance. We aimed to randomise 30 participants 2:1 to receive intranasal inoculation with intervention or control given inoculation with 10^4^-10^5^ CFU previously resulted in ~40-50 % colonisation.^9,11^ For Study B, we aimed to recruit ten Nlac-colonised participants to each group (sample size and power calculations outlined in Supplementary Appendix 1).

Statistical analyses were performed using GraphPad Prism software, version 8·0 (GraphPad Software Inc.). Normality tests were performed using Shapiro-Wilk. Parametrically-distributed and non-parametrically-distributed data were summarised with mean (+/- SD) and median (+/- range or interquartile range [IQR]), respectively. Non-parametric paired data were analysed using the two-tailed Wilcoxon matched pairs signed rank test, or the Friedman test, with Dunn’s multiple comparisons post-test when assessing more than two groups. Unpaired data were analysed using the Mann-Whitney test, or the Kruskal-Wallis test with Dunn’s multiple comparisons post-test when assessing more than two groups. Correlation analyses for non-parametric data were performed using Spearman’s Rho (*r*_s_). Proportions between two groups were assessed using Fisher’s exact test. All *P* values were two-tailed, and *P* values ≤ 0·05 were considered statistically significant. A data monitoring committee was not utilised for either study. In line with guidance^17^, we utilised complete case analysis. Multiple imputation methods were not utilised as data were missing for < 5 % of the total dataset and were missing completely at random (biological samples were not available for *n* = 3/216 (1·4 %) immunological assays (PBMC or plasma) and *n* = 2/189 (1·05 %) microbiological assays (throat swab or nasal wash).

Both trials are registered with *ClinicalTrials.gov* (NCT03633474 and NCT03549325, respectively) and are now closed to new participants.

#### Role of the Funding Source

The funders of the studies had no role in study design, data collection, data analysis, data interpretation, or writing of the report. The corresponding author had full access to the data in the study and had final responsibility for the decision to submit for publication.

## Results

**Figure 1:**
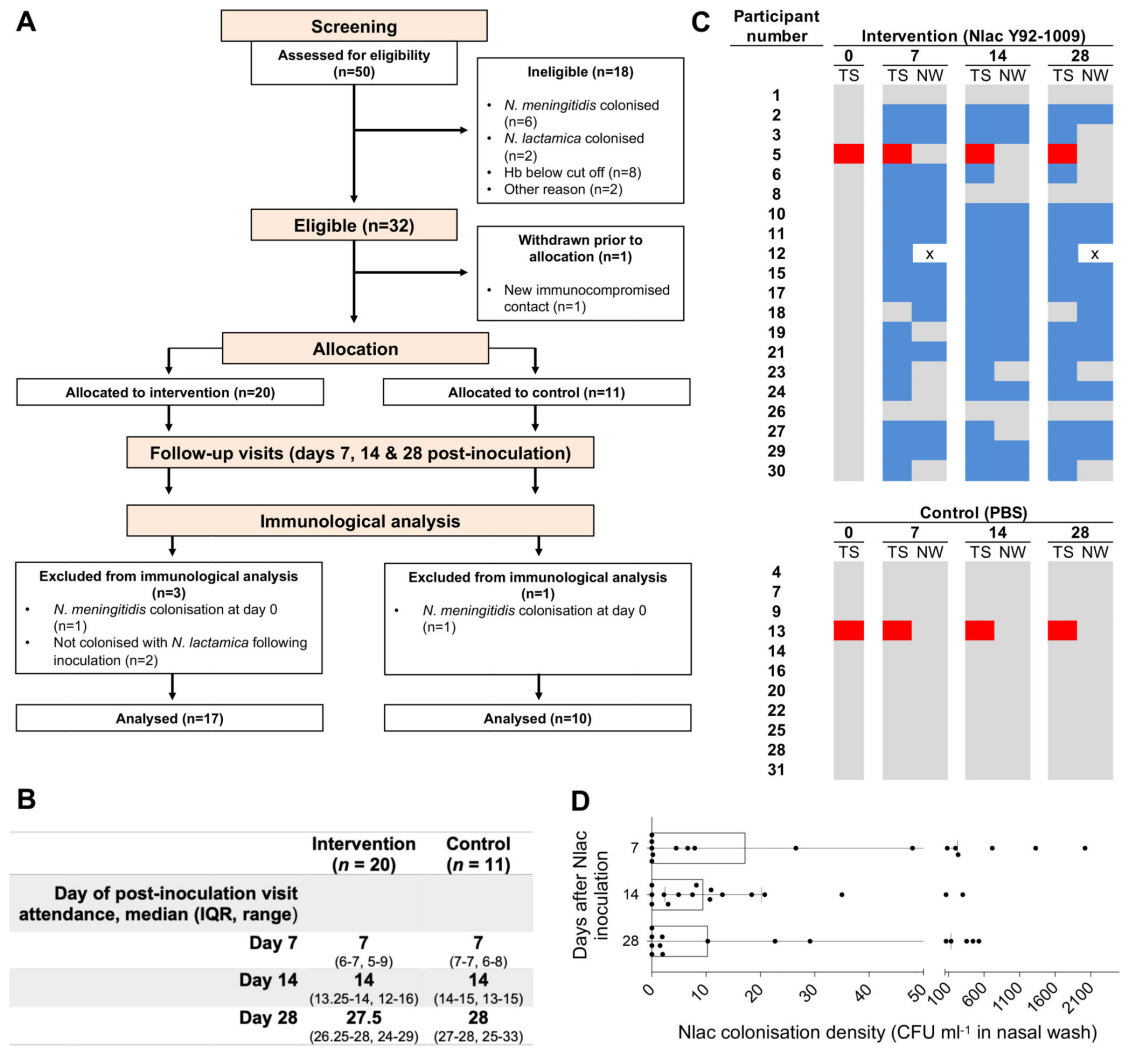
Controlled human infection model experiment using *N. lactamica* Y92-1009 (Study A). (**A**) Study flow diagram showing allocation to intervention (Nlac) or control (PBS) groups, study completion, and participants included in the immunological analyses. Hb − haemoglobin concentration in whole blood. (**B**) Timing of study visits amongst participants assigned to intervention and controls groups. (**C**) Pattern of *Neisseria* species colonisation amongst participants as determined by culture of oropharyngeal throat swab (TS) and nasal wash (NW) specimens. Days after inoculation at the time of sampling are shown in bold. Colour blocks represent no Nlac or Nmen cultured (grey), Nlac cultured (blue), Nmen cultured (red), and absence of nasal wash sample for culture (white with black cross). (**D**) Nlac colonisation densities (CFU ml’^1^ in nasal wash) amongst Nlac-colonised participants at post-inoculation time points. Columns and error bars represent median and IQR, respectively.

For Study A, *n* = 31/50 participants assessed for eligibility between Sep 5, 2018, and Mar 3, 2019, were randomised 2:1 to intra-nasal inoculation with Nlac (*n* = 20, median inoculum size of 0·93 x 10^5^ CFU, range 0·4-3·4 x10^5^) or PBS (*n* = 11) (Figure 1A). Baseline characteristics (Table 1), and timing of post-inoculation study visits (Figure 1B), were similar between groups. No participants were lost to follow up and all participants completed the study per-protocol. *n* = 17/20 Nlac-inoculated participants became colonised. No PBS-inoculated participants became Nlac colonised (Figure 1C). Participant eight lost Nlac colonisation following ciprofloxacin for a urinary tract infection between day 7-14 post-inoculation. No other participants received antibiotics. Two participants acquired Nmen colonisation following screening. Nlac colonisation density was similar across timepoints (Figure 1D) and there was no association between returned nasal wash volumes and total Nlac CFU (*r_s_* -0.1089, *P* = 0.47). Symptoms consistent with possible URT infection including cough, sore throat and rhinorrhoea occurred in *n* = 10/20 Nlac-inoculated and *n* = 6/11 PBS-inoculated participants between day 7-28 post-inoculation (*P* > 0·99, Fisher’s exact test), with similar URT symptom reporting frequencies suggesting that these symptoms were likely unrelated to Nlac and more likely due to URT viral infections or other non-infective causes. There were three additional adverse events, two of which were unrelated to the intervention, and one which was related but of no consequence (participant snorted during the intra-nasal inoculation procedure causing nasal secretion to run into the eye). There were no serious adverse events (Supplementary Table 4).

For Study B, *n* = 21/33 participants assessed for eligibility between Mar 3, 2017, and Apr 14, 2020, received intra-nasal inoculation with Nlac (median inoculum size of 1·47 x 10^5^ CFU, range 0·78-5·12 x10^5^). The median age of participants was 31 years (range 25-45) (76 % female and 24 % male). Nlac colonisation was detected in *n* = 6/7 and *n* = 7/13 of participants assigned to receive colonisation-eradicating ciprofloxacin at 14 days and 4 days, respectively, following Nlac inoculation, and *n* = 19 participants completed the study per-protocol(Supplementary Figure 1). Unfortunately, recruitment to Study B ceased due to the COVID-19 pandemic before the predetermined sample size (*n* = 20) of Nlac-colonised participants was recruited. A pragmatic decision was made to terminate the study early following an interim analysis of immunological data with six Nlac-colonised participants in each group. Administration of ciprofloxacin was associated with loss of Nlac colonisation in 100 % of cases. Symptoms consistent with possible URT infection occurred in *n* = 5/21 participants. There were eight additional adverse events, all of which were unlikely related to the intervention, and there were no serious adverse events (Supplementary Table 5).

Amongst Study A participants, frequencies of B_PLAS_ and B_MEM_ were measured using ELISpot assays (Supplementary Figure 2A-B, and 3A-B). Given B_PLAS_ occur transiently in blood following vaccination or natural infection,^18,19^ peak IgA- and IgG-secreting B_PLAS_ frequencies, occurring on day 7, 14 or 28 post-inoculation, were compared to the baseline frequency. As vaccine-induced antigen-specific B_MEM_ do not circulate until 14-28 days following vaccination,^18,20^ IgG B_MEM_ frequencies were compared at baseline versus day 28. B_MEM_ frequencies were also assessed at 7 and 14 days post-inoculation to study dynamics.

**Figure 2:**
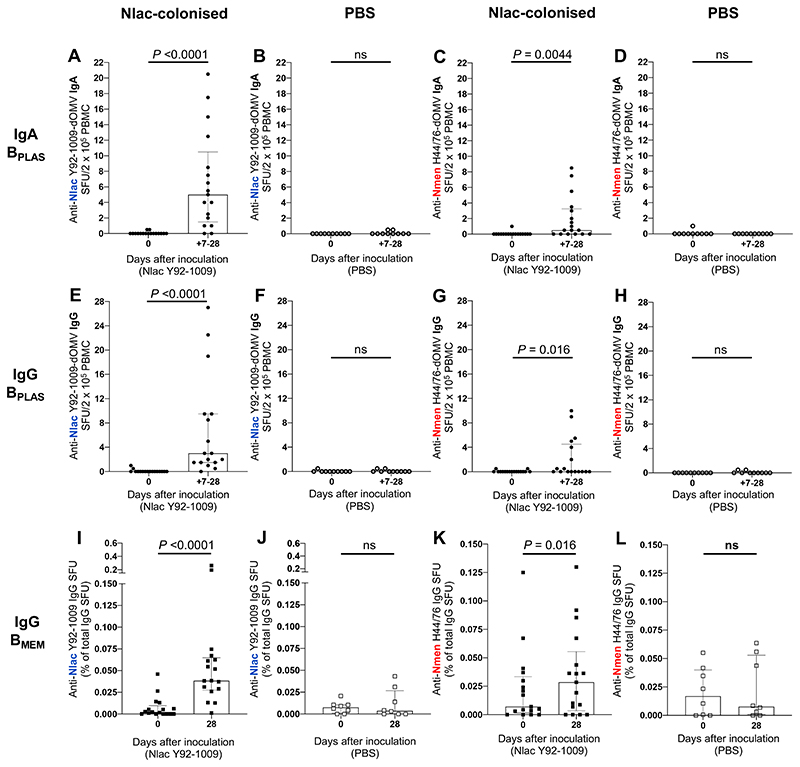
Colonisation with *N. lactamica* induces *N. lactamica-* and *N. meningitidis-specific* IgA- and IgG-secreting plasma cells and IgG memory B cells. PBMC isolated from whole blood at baseline (day 0) and on days 7, 14 and 28 post-inoculation were derived from Nlac-colonised (filled circles) and PBS-inoculated (hollow circles) participants and assessed by ELISpot for the presence of IgA-secreting (**A**-**D**) and IgG-secreting (**E-H**) B_PLAS_ with specificity to Nlac Y92-1009-dOMV (**A, B, E, F**) or Nmen H44/76-dOMV (**C, D, G, H**). B_PLAS_ were visualised as spot-forming units (SFU), with mean SFU derived from experimental duplicates having adjusted for non-antigen-specific SFU by subtracting the mean SFU enumerated in keyhole limpet hemocyanin-coated membranes. For each participant, the highest number of SFU per 2 x 10^5^ PBMC is shown (between days 7-28) for each antigen. For IgG B_MEM_ responses, PBMC from day 0 and 28 days post-inoculation derived from Nlac-colonised (filled squares) and PBS-inoculated (hollow squares) participants were thawed and polyclonally stimulated prior to assessment by ELISpot for the presence of IgG-secreting cells with specificity to Nlac Y92-1009-dOMV (**I-J**) or Nmen H44/76-dOMV (**K-L**). Mean IgG SFU were derived from experimental triplicates having adjusted for non-specific SFU by subtracting the mean SFU enumerated in KLH-coated membranes. Antigen-specific IgG SFU are shown as a percentage of the total number of IgG-secreting SFU. Columns and error barsindicate median and IQR, respectively. SFU frequencies compared using the Wilcoxon matched-pairs signed rank test (ns − not significant, *P* >0·05) (*n* = 17 Nlac-colonised participants, *n* = 10 PBS-inoculated participants. Note: PBMC extraction failed for *n* = 2/10 participants inoculated with PBS at day 28, therefore only *n* = 8 participants in B_MEM_ analysis).

Amongst Nlac-colonised participants there was a significant increase in the frequency of IgA-and IgG-secreting B_PLAS_ with specificity to both Nlac-dOMV (hereafter, Nlac-specific B_PLAS_) and Nmen-dOMV (hereafter, Nmen-specific B_PLAS_) (Figure 2A,C,E and G). Where Nlac-specific B_PLAS_ increased post Nlac-colonisation, peak frequencies occurred in *n* = 2/15 on day 7, *n* = 12/15 on day 14, and *n* = 1/15 on day 28 for IgA-secreting B_PLAS_, and *n* = 4/16 on day 7, *n* = 12/16 on day 14, and *n* = 2/16 on day 28 for IgG-secreting B_PLAS_. There was no change in the frequency of B_PLAS_ detected in TT-coated wells (Supplementary Figures 2G and 2I), excluding bystander activation of the B_MEM_ pool. Amongst PBS-inoculated participants, Nlac-, Nmen- or TT-specific B_PLAS_ did not increase (Figure 2B,D,F and H, and Supplementary Figure 2H and J). SFU enumerated in KLH-coated wells were consistently low in frequency for both groups (Supplementary Figure 2C-F).

Amongst Nlac-colonised participants there was also a significant increase in the percentage of the total number of IgG B_MEM_ (hereafter, percentage of B_MEM_) with specificity to both Nlac-dOMV (hereafter, Nlac-specific B_MEM_) and Nmen-dOMV (hereafter, Nmen-specific B_MEM_) at day 28 compared to baseline (Figure 2I,K). There was no change in the percentage of IgG B_MEM_ with specificity to Flu HA (hereafter, Flu HA-specific B_MEM_), the positive control antigen (Supplementary Figure 3G). Amongst PBS-inoculated participants, Nlac- or Nmen- and Flu HA-specific B_MEM_ frequencies did not increase (Figure 2J and L, and Supplementary Figure 3H). IgG SFU enumerated in KLH- and anti-IgG mAb-coated wells was similar across timepoints indicating B_MEM_ ELISpot assay performance stability (Supplementary Figure 3C- F,I-J).

Peak frequencies of IgG- and IgA-secreting B_PLAS_ specific to Nlac and Nmen significantly positively correlated (Supplementary Figure 4A and B). In addition, peak frequencies of Nlac-and Nmen-specific B_PLAS_ significantly positively correlated for both IgG- and IgA-secreting B_PLAS_ (Supplementary Figure 4C-D), with the timing of peak Nmen-specific B_PLAS_ frequencies coinciding with peak Nlac-specific B_PLAS_ frequencies in the majority (*n* = 9/11 and *n* = 8/8 of cases for IgA- and IgG-secreting B_PLAS_, respectively), suggesting that the timing and magnitude of Nlac- and Nmen-specific B_PLAS_ responses induced by Nlac colonisation were interdependent. However, Nlac-specific B_PLAS_ response frequencies were universally higher than those specific to Nmen (median increase in Nlac-specific and Nmen-specific B_PLAS_ per 2 x 10^5^ PBMC of 5 [IQR 1·5-10·3] vs. 0·5 [0.0-3·3] for IgA-secreting B_PLAS_ [*P* = 0·0010, Wilcoxon test], and 2 [1·0-9·5] vs. 0 [0·0-4·5] for IgG-secreting B_PLAS_ [*P* = 0·0001]). Median increases in IgG B_MEM_ responses, expressed as a percentage of the total IgG B_MEM_ population, were also higher for responses specific to Nlac (0·0260 %, IQR 0·0125-0·0629, median 16.3-fold increase) vs. Nmen (0·0044 %, 0.0000-0·0165, median 4·0-fold increase) (*P* = 0·0020). There was no significant correlation between: (i) Nlac- and Nmen-specific B_MEM_ frequencies atbaseline (Supplementary Figure 5A) or, (ii) increases in the frequency of Nlac- and Nmen-specific B_MEM_ following Nlac-colonisation (Supplementary Figure 4E).

**Figure 3:**
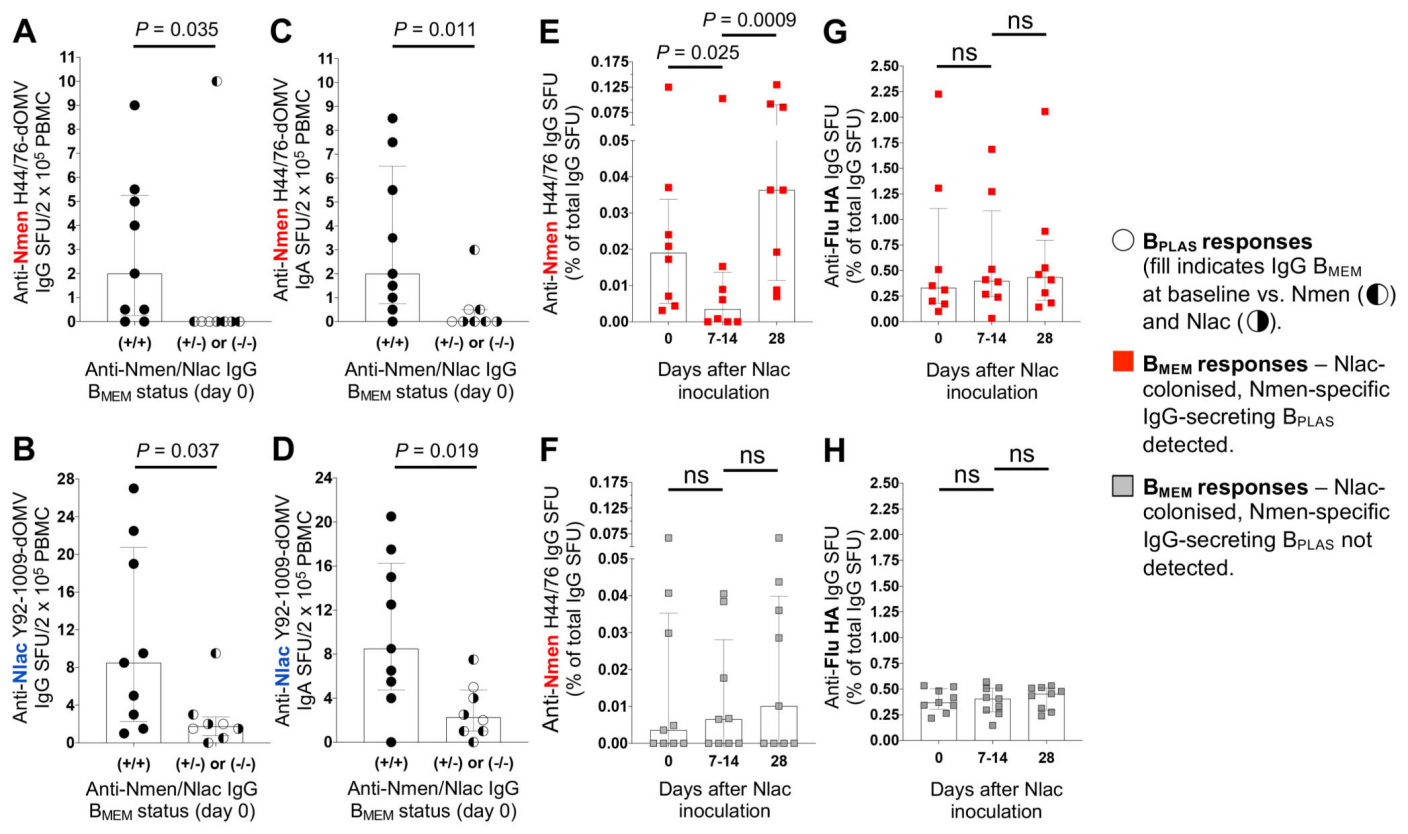
*N. lactamica-* and *N. meningitidis-specific* plasma cell response frequencies are associated with baseline memory B cell status. IgG-secreting B_PLAS_ response frequencies (circles) with specificity to Nmen H44/76 dOMV (**A**) or Nlac Y92-1009-dOMV (**B**) amongst Nlac-colonised participants with (+) or without (-) detectable Nmen H44/76-dOMV-specific (left-filled circles) and Nlac Y92-1009 dOMV-specific (right-filled circles) IgG B_MEM_ responses detectable in blood at baseline, as determined using the IgG B_MEM_ ELISpot assay *(n* = 17). IgA-secreting B_PLAS_ response frequencies (circles) with specificity to Nmen H44/76-dOMV (**C**) or Nlac Y92-1009-dOMV (**D**). IgG B_MEM_ frequencies with specificity to Nmen H44/76-dOMV amongst participants with (red squares) (**E**) or without (grey squares) (**F**) detectable Nmen H44/76-dOMV-specific IgG-secreting B_PLAS_ responses following Nlac colonisation, comparing day-0 and day-28 B_MEM_ frequencies with the lowest B_MEM_ frequency measured on day 7 or 14. Anti-influenza antigen reagent 09/174, H1N1 (Flu HA)-specific IgG B_MEM_ frequencies in the same groups (**G, H**). Columns and error bars indicate median and IQR, respectively. SFU frequencies compared with the Mann-Whitney test (**A**-**D**) or the Kruskal-Wallis test with Dunn’s multiple comparisons test (**E**-**H**) (ns - not significant, *P* > 0·05).

Antigen-specific B_PLAS_ frequencies are higher following boosting compared with priming after intra-muscular vaccination,^18,19^ so we tested the hypothesis that variation in Nmen-specific B_PLAS_ frequencies are due to recall of pre-existing IgG B_MEM_ responses. We reasoned that Nmen-specific IgG B_PLAS_ responses would occur more frequently, and would be higher in magnitude, amongst participants with cross-reactive B_MEM_ responses at baseline. We compared Nmen-specific IgG-secreting B_PLAS_ responses amongst Study A participants with and without both detectable Nlac- and Nmen-specific B_MEM_ responses at baseline (Figure 2, I and K, and Supplementary Figure 6). Nmen-specific IgG-secreting B_PLAS_ responses were detected more often (Fisher’s exact test *P* = 0·015) and were higher in magnitude (Figure 3A) amongst participants where both Nlac- and Nmen-specific B_MEM_ responses were detected at baseline. Interestingly, Nlac-specific IgG-secreting B_PLAS_ response frequencies (Figure 3B) and Nmen- and Nlac-specific IgA-secreting B_PLAS_ frequencies (Figure 3, C and D) were also significantly higher amongst this group. Further evidence that Nlac colonisation induced recall of pre-existing B_MEM_ responses was provided by assessment of the dynamics of Nmen-specific B_MEM_ frequencies. Amongst Nlac-colonised participants with detectable Nmen-specific IgG-secreting B_PLAS_ responses, Nmen-specific B_MEM_ frequencies decreased significantly from baseline to the day 7-14 nadir, suggesting differentiation of pre-existing Nmen-specific B_MEM_ into B_PLAS_, before increasing from nadir to day 28, suggesting subsequent expansion of the B_MEM_ pool (Figure 3E). The same dynamics were not observed amongst Nlac-colonised participants where Nmen-specific IgG-secreting B_PLAS_ were undetectable (Figure 3F). Importantly, Flu HA-specific B_MEM_ frequencies from matched time points remained stable in both groups (Figure 3, G and H).

**Figure 4:**
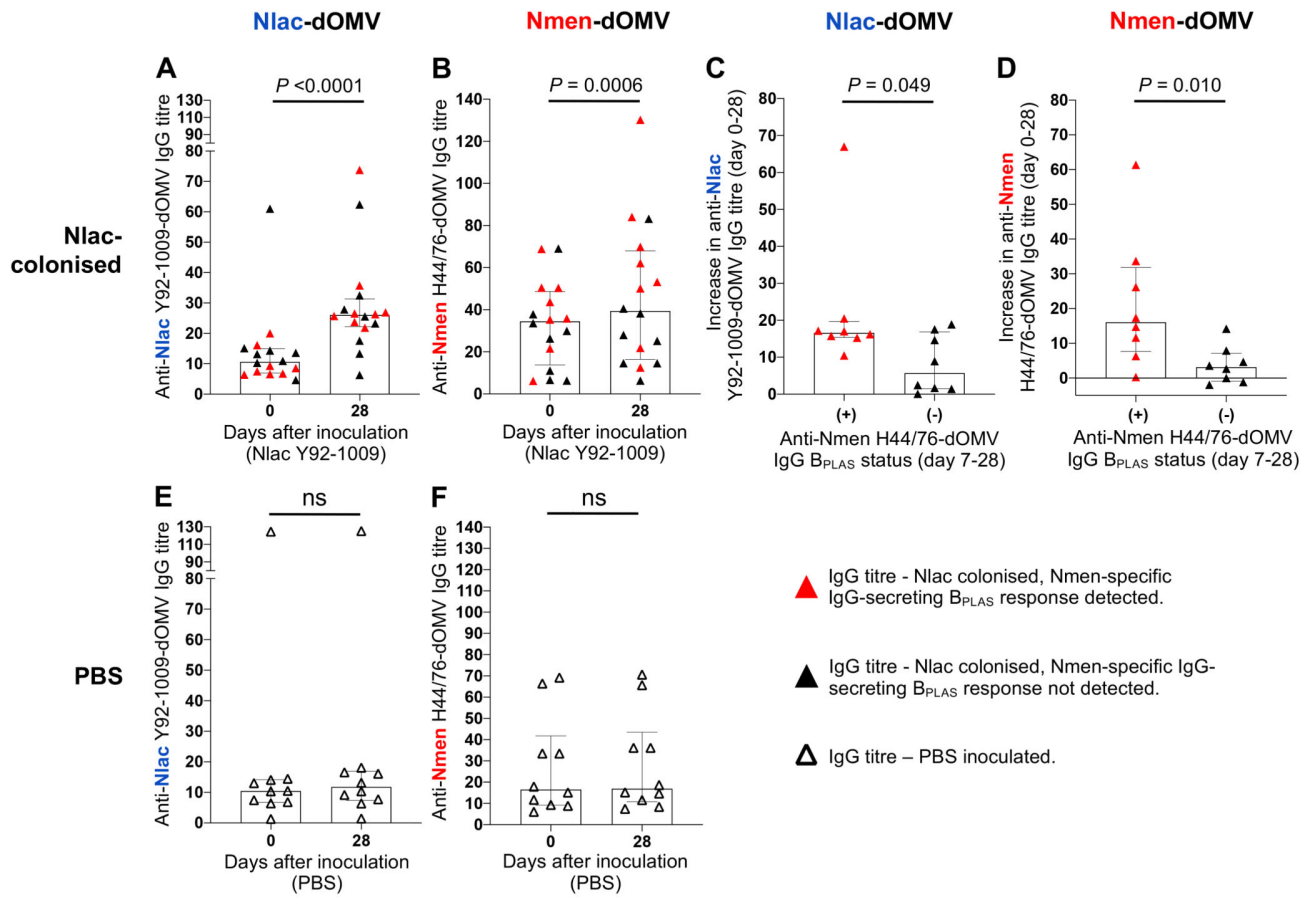
Colonisation with *N. lactamica* induces *N. lactamica-* and *N. meningitidis-specific* IgG in plasma. Anti-Nlac Y92-1009-dOMV IgG titres in plasma obtained at baseline (day 0) and day 28 post-inoculation from participants who were Nlac-colonised (**A**) or PBS-inoculated (**E**). Anti-Nmen H44/76-dOMV IgG titres in plasma obtained at baseline and day-28 post-inoculation from participants who were Nlac-colonised (**B**) or PBS-inoculated (**F**). Increase in anti-Nlac Y92-1009-dOMV (**C**) and anti-Nmen H44/76-dOMV (**D**) IgG titres between baseline and day 28 amongst Nlac-colonised participants with (+, red triangles) versus without (-, black triangles) detectable Nmen-specific IgG B_PLAS_ responses post-inoculation. Columns and error bars indicate median and IQR, respectively. IgG titres compared using the Wilcoxon matched-pairs signed rank test (**A**, **B**, **E**, **F**) or the Mann-Whitney test (**C**-**D**) (ns - not significant, *P* > 0·05) (*n* = 16 Nlac-colonised participants, *n* = 10 PBS-inoculated participants. Note: plasma was not available at day 0 for *n* = 1/17 Nlac-colonised participants).

Anti-Nlac-dOMV IgG (anti-Nlac IgG) titres, measured using ELISA (Supplementary Figure 7), increased significantly from baseline to day 28 post-inoculation amongst Nlac-colonised participants in Study A (Figure 4A). A smaller but significant rise in anti-Nmen-dOMV IgG (anti-Nmen IgG) titre was also observed (Figure 4B). In keeping with previously-outlined B_PLAS_ dynamics, increases in anti-Nlac and anti-Nmen IgG titres between day 0-28 significantly positively correlated (Supplementary Figure 4F). There was no change in either IgG titres amongst PBS-inoculated participants (Figure 4, E and F). Increases in IgG titres between day 0-28 significantly correlated with peak IgG-secreting B_PLAS_ frequencies for both Nlac- and Nmen-specific responses (Supplementary Figure 4G and H). Furthermore, increases in anti-Nlac and anti-Nmen IgG titres between day 0-28 were higher amongst Nlac-colonised participants where Nmen-specific IgG B_PLAS_ responses were detected post-inoculation (Figure 4C and D). At baseline, significant positive correlations were observed between: (i) anti-Nlac and anti-Nmen IgG titres (Supplementary Figure 5B), and (ii) anti-Nmen (but not anti-Nlac) IgG titres and Nmen-specific B_MEM_ frequencies (Supplementary Figure 5C and D).

**Figure 5:**
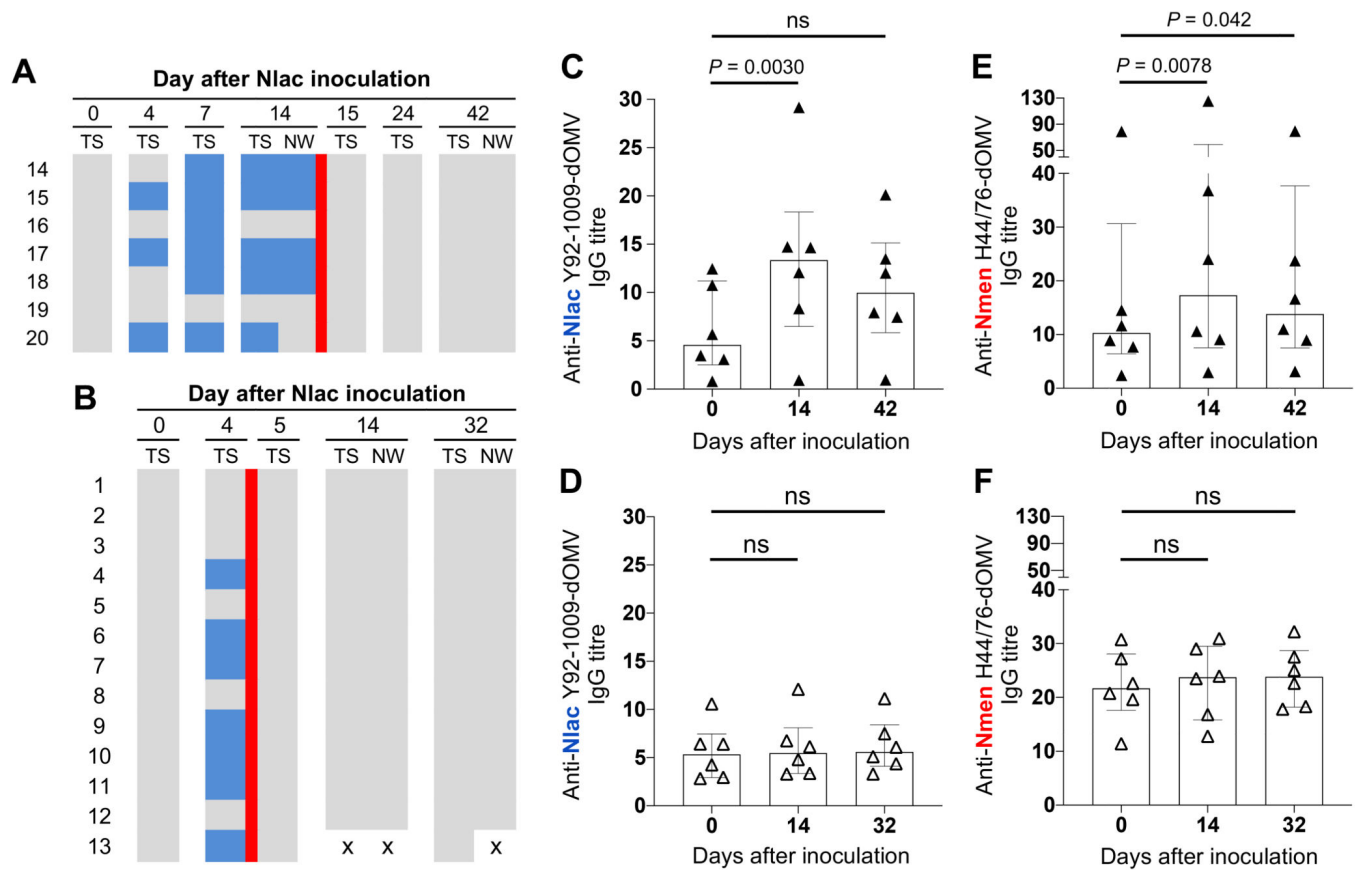
Induction of IgG with specificity to *N. lactamica* and *N. meningitidis* is dependent on the duration of *N. lactamica* colonisation. Study B participants were inoculated with 10^5^ CFU Nlac with *Neisseria* species colonisation status determined by culture of oropharyngeal throat swab (TS) and nasal wash (NW) specimens, as indicated. Colour blocks represent no Nlac or Nmen cultured (grey), Nlac cultured (blue), Nmen cultured (red), and absence of sample for analysis (white with black cross). Nlac colonisation was eradicated following 14 days (**A**) or 4 days (**B**) of Nlac colonisation by administration of single-dose oral ciprofloxacin, as indicated (red line). Serum was obtained at baseline (day 0), 14 days post-inoculation, and at 28 days following ciprofloxacin therapy and probed for IgG with specificity to Nlac Y92-1009-dOMV (**C**-**D**) and Nmen H44/76-dOMV (**E**-**F**) amongst participants colonised with Nlac for 14 days (closed triangles) versus 4 days (open triangles). Columns and error bars represent median and IQR, respectively. IgG titres compared using the Friedman test with Dunn’s multiple comparisons test (ns - not significant, *P* > 0.05) (*n* = 6 Nlac-colonised participants in each group).

We reasoned that the induction of an adaptive immune response during Nlac colonisation would be influenced by colonisation duration. In Study B, we measured anti-Nlac and anti-Nmen IgG in serum at both 14 days post-inoculation, and 28 days following eradication with ciprofloxacin, amongst participants colonised with Nlac for 14 versus 4 days (Figure 5, A and B). Amongst participants colonised with Nlac for 14 days, there was a significant increase in both anti-Nlac and anti-Nmen IgG titres, when comparing day 14 with baseline. At 28 days post-eradication, anti-Nlac IgG titres were no longer significantly higher than baseline (*P* = 0·087) but anti-Nmen IgG titres were (Figure 5, C and E). Interestingly, amongst participants colonised with Nlac for only 4 days, anti-Nlac and anti-Nmen IgG titres did not increase (Friedman ANOVA *P* = 0·14 and *P* = 0·14, respectively) (Figure 5, D and F).

Post-hoc analyses were performed comparing Nlac colonisation density with B cell and IgG responses amongst Nlac-colonised participants in Study A. We observed no significant correlations between Nlac-specific B_MEM_ at baseline and Nlac colonisation density measured at 7-28 days (Supplementary Figure 8A-C). Although the percentage of Nmen-specific B_MEM_ did not correlate with Nlac colonisation density at 7 days following Nlac inoculation, inverse correlations were observed at 14 and 28 days (Supplementary Figure 8D-F). Given the positive correlations between baseline frequencies of Nmen-specific B_MEM_ and day-28 anti-Nlac and anti-Nmen IgG titres (Supplementary Figure 5E and F), we compared IgG titres with Nlac colonisation densities on day 28. While there were no significant correlations between Nlac colonisation on day 7 and baseline anti-Nlac IgG titres (Supplementary Figure 9A), significant negative correlations were observed between day-28 Nlac colonisation densities and (i) anti-Nlac IgG titres (Supplementary Figure 9B), and (ii) the increase in anti-Nlac IgG titres between day 0-28 (Supplementary Figure 9C). There were no significant associations between anti-Nmen IgG titres and Nlac colonisation density (Supplementary Figure 9, D-F). Next, we looked for correlations between B_PLAS_ responses and Nlac colonisation density, opting to assess correlations between B_PLAS_ peaks and Nlac colonisation density at 14 days and 28 days post-inoculation given the timing of peak B_PLAS_ responses. No significant correlations were observed at day 14 but at day 28 there was a significant negative correlation between Nlac colonisation density and peak Nlac- and Nmen-specific IgA-secreting B_PLAS_ frequencies (Supplementary Figure 10A and C). There were no significant correlations between Nlac colonisation density and Nlac- or Nmen-specific IgG-secreting B_PLAS_ frequencies (Supplementary Figure 10B and D).

## Discussion

This work demonstrated that asymptomatic pharyngeal colonisation with Nlac induces adaptive humoral and B cell responses cross-reactive with Nmen, including formation of cross-reactive immunological memory. This supports the hypothesis that the protective effect of Nlac on Nmen colonisation is due to cross-reactive adaptive immune responses.

Expansion of Nmen-specific IgA- and IgG-secreting B_PLAS_ was not universal but was observed in 65 % and 47 % of Nlac-colonised participants, respectively, while Nmen-specific B_MEM_ frequencies increased in 53 %. These data are consistent with a previous Nlac CHIME where ~65 % of Nlac-colonised participants developed anti-Nmen OPA.^11^ In contrast, expansion of Nlac-specific B cell responses was almost universal (increases in IgA- and IgG-secreting B_PLAS_ and IgG B_MEM_ in 88 %, 94 % and 94 %, respectively) with responses significantly higher in magnitude compared to Nmen-specific responses. These differences are likely explained by amino acid divergence amongst orthologous surfaced-expressed proteins found across the two species.^21^ While definitive protein antigens responsible for the cross-reactive B cell responses remain undefined, previous analyses of Nlac Y92-1009-dOMV using mass spectrometry revealed 15 protein orthologues present amongst pathogenic *Neisseriaceae*, while probing of Nmen H44/76-dOMV with anti-sera raised in mice following Nlac Y92-1009-dOMV vaccination by Western blotting revealed at least 14 cross-reactive bands.^21^

Development of novel strategies to protect against Nmen colonisation are justified as current protein-based serogroup B Nmen vaccines do not impact on this critical outcome.^4–6^ We propose that colonisation with Nlac could be utilised as an immunobiotic intervention to prevent Nmen colonisation given that: (1) Nlac colonisation protects against Nmen colonisation,^9^ and (2) Nlac colonisation density negatively correlated with B_PLAS_ and IgG responses observed in Study A, suggesting a role for adaptive immune responses generated in response to URT Nlac colonisation in controlling colonisation. The immunological mechanisms underlying this effect are unknown but could be due to induction of agglutinating IgA/IgG at the mucosal surface,^22,23^ or neutrophil associated clearance secondary to IL-17 secretion by antigen-specific CD4+ T cell of Th17 effector phenotype.^24–26^

We predict that future prophylactic use against Nmen will be incomplete if Nlac is utilised in its current wild-type form as cross-reactive immune responses to Nmen were not universal. Furthermore, immune dynamics were consistent with a recall immune response (Figure 3). However, induction of cross-reactive immunity may not be the only contributory mechanism. Colonisation with Nlac following inoculation is less likely in Nmen-colonised individuals,suggesting competition for niche occupation.^9^ This may explain why Study A participant five failed to colonise with Nlac following inoculation. We do not anticipate that this phenomenon would significantly impact the use of Nlac as an immunobiotic intervention as this strategy would probably most effectively be used in childhood, prior to the natural upswing in Nmen colonisation acquisition during adolescence, to enable maximal protection to be afforded against Nmen transmission.^1^

Notwithstanding the possible multifactorial nature of the protective effect of Nlac on Nmen, it would be optimal if colonising Nlac induced broad anti-Nmen mucosal immunity resulting in Nmen colonisation protection and protection against Nmen disease though generation of anti-Nmen SBA. In this regard, in a recent first-in-man study we demonstrated that colonisation with genetically-modified Nlac (GM-Nlac) expressing the meningococcal vaccine antigen Neisseria adhesin A (NadA), induced NadA-specific adaptive responses and anti-Nmen SBA.^15^ With proof of principle and safety established, we now aim to enhance and broaden anti-Nmen adaptive immunity generated using GM-Nlac, through co-expression of other meningococcal vaccine antigens.

The randomised, placebo-controlled design of Study A was its key strength. Limitations should be discussed. Firstly, for Study A, the sample size was informed using Nlac IgG titres. While this sample size was sufficient to investigate Nlac- and Nmen-specific B cell responses, it only enabled an exploratory analysis of associations between B_MEM_/B_PLAS_ responses and Nlac colonisation density. Secondly, the assessment of Nlac colonisation density in nasal wash was exploratory; colonisation density sampled using oropharyngeal swabs was not measured, precluding comparison of Nlac colonisation density between nasal wash and oropharyngeal swab. Thirdly, we did not assess responses restricted to the mucosal compartment, focusing instead on responses in blood. The key limitation of Study B was that due to the COVID-19 pandemic recruitment was terminated early. However, this should not detract from the main result, that seroconversion only occurred amongst participants assigned to Nlac colonisation for 14 days.

## Supplementary Material

Supplementary Material

## Figures and Tables

**Table 1:** Baseline characteristics.

	Intervention (n = 20)	Control (n = 11)
**Age** (years)	**30** (23.3-34.8)	**25** (21.0-29.0)
**Female**	**9** (45)	**7** (64)
**Male**	**11** (55)	**4** (36)

Data are median (range), *n* (%).

## Data Availability

All data associated with these studies are present in the paper, associated Clinical Study Protocols and Supplementary Materials. Individual deidentified participant data that underlie the results reported in this article, including data dictionaries, will be shared with investigators whose proposed use of the data has been approved by an independent review committee identified for this purpose, and following appropriate institutional and ethical review. Data sharing will be permitted to achieve the aims in the approved proposal. Proposals should be directed to the corresponding author (a.p.dale@soton.ac.uk); to gain access, data requestors will need to sign a data access agreement. Data will be available beginning 1 month and ending 24 months following article publication.

## Research in context

### Evidence before this study

Future vaccines against meningococcal, pneumococcal and *Haemophilus influenzae* disease, suitable for mass deployment in resource poor countries, could be low-cost strategies which induce herd protection against acquisition of these pathogens in the upper respiratory tract. One potential strategy could be to exploit naturally-occurring protective relationships between pathogenic and commensal organisms. In nature, an inverse epidemiological relationship exists between colonisation with the harmless commensal, *Neisseria lactamica*, and *Neisseria meningitidis-associated* colonisation and disease, with circumstantial evidence suggesting that this phenomenon may be due to induction of cross-reacting immunity. If the nature of this cross-reactive immunity was understood, it could be exploited to develop vaccines that induced herd protection against *N. meningitidis.* Specifically, this knowledge could inform the development of novel serogroup B meningococcal vaccines where current formulations provide no protection against colonisation. We searched PubMed for studies published before 16/02/2022, with no language restrictions, using the search terms *Neisseria lactamica* AND *Neisseria meningitidis* AND (colonisation OR carriage) AND (humoral OR antibody OR B cell OR T cell OR protection). This identified 36 studies, of which two were controlled human infection studies designed to assess the effect of *N. lactamica* colonisation on (i) *N. meningitidis* colonisation, and (ii) the induction of anti-*N. meningitidis* adaptive immunity. In the first study (n = 310), controlled infection with *N. lactamica* led to a significant reduction in *N. meningitis* colonisation, with an effect observed by two weeks and persisting over at least 16 weeks. In the second study (*n*=61), *N. lactamica* colonisation was associated with the induction of anti-*N. meningitidis* opsonophagocytic activity but not serum bactericidal activity.

### Added value of this study

In this study, using deliberate infection of human volunteers, we demonstrate for the first time that colonisation with *N. lactamica* induces anti-*N. meningitidis* IgG and B cell responses, including the formation of immunological memory, with seroconversion dependent upon the duration of colonisation. In addition, we outline B cell dynamics that support the notion that *N. meningitidis*-specific plasma cell responses induced by *N. lactamica* colonisation are due to recall of pre-existing cross-reactive immunological memory. Furthermore, we reveal significant negative correlations between anti-Nlac IgG titers and IgA-secreting plasma cell frequencies induced by *N. lactamica* colonisation and *N. lactamica* colonisation density, suggesting a role for these responses in bioburden control.

### Implication of all the available evidence

Our findings support the hypothesis that the protective effect of *N. lactamica* colonisation on *N. meningitidis* colonisation and disease is due to induction of cross-reactive adaptive immune responses. The observation that *N. meningitidis-specific* plasma cell and memory B cell responses were not always generated amongst *N. lactamica*-colonised participants is in keeping with previous work demonstrating that protection afforded by *N. lactamica* colonisation on *N. meningitidis* colonisation was not universal. Future studies will assess whether induction of anti-*N. meningitidis* adaptive immune responses by *N. lactamica* prevents subsequent *N. meningitidis* colonisation, and whether this activity can be enhanced through genetic modification of *N. lactamica* to express meningococcal vaccine antigens. Ultimately this will lead to low-cost live bacterial vectored vaccines suitable for use in regions with high rates of meningococcal disease.
