# Supplementary material for: Effect of colonisation with Neisseria lactamica on cross-reactive anti-meningococcal B cell responses: A randomised, controlled, human infection trial
Source: Lancet Microbe. Author manuscript; Available in PMC 2023 Sep 8. (PMC7615047; doi:10.1016/S2666-5247(22)00283-X)
Supplement: Supplementary Material [file EMS185183-supplement-Supplementary_Material.pdf]

Supplementary Material

Table of contents

Supplementary Figure 1. Participant flow diagram (Study B). Page 36

Supplementary Figure 2. Representative B_{PLAS} ELISpot images and pooled KLH-specific and TT-specific B_{PLAS} responses amongst participants. Page 37

Supplementary Figure 3. Representative IgG B_{MEM} ELISpot images and pooled KLH-specific and Flu HA-specific IgG B_{MEM} responses amongst participants. Page 38

Supplementary Figure 4. Associations between *N. lactamica*- and *N. meningitidis*-specific humoral and B cell responses following *N. lactamica* colonisation. Page 40

Supplementary Figure 5. Associations between *N. lactamica*- and *N. meningitidis*-specific humoral and B cell responses at baseline. Page 42

Supplementary Figure 6. IgG B_{MEM} ELISpot data for *N. lactamica*-colonised participants at baseline. Page 43

Supplementary Figure 7. Representative plasma IgG ELISA data for a single *N. lactamica*-colonised participant. Page 44

Supplementary Figure 8. Baseline *N. meningitidis*-specific IgG B_{MEM} frequencies inversely correlate with *N. lactamica* colonisation density. Page 45

Supplementary Figure 9. *N. lactamica*-specific IgG titres inversely correlate with *N. lactamica* colonisation density. Page 46

Supplementary Figure 10. *N. lactamica*-specific IgA-secreting B_{PLAS} frequencies inversely correlate with *N. lactamica* colonisation density. Page 47

Supplementary Table 1. Study A clinical procedures. Page 48

Supplementary Table 2. Study B clinical procedures. Page 49

Supplementary Table 3. Inclusion and exclusion criteria for the *N. lactamica* (Y92-1009) CHIMEs. Page 50

Supplementary Table 4. Study A adverse events. Page 51

Supplementary Table 5. Study B adverse events. Page 52

Supplementary Appendix 1. Sample size and power calculation for Study B. Page 53

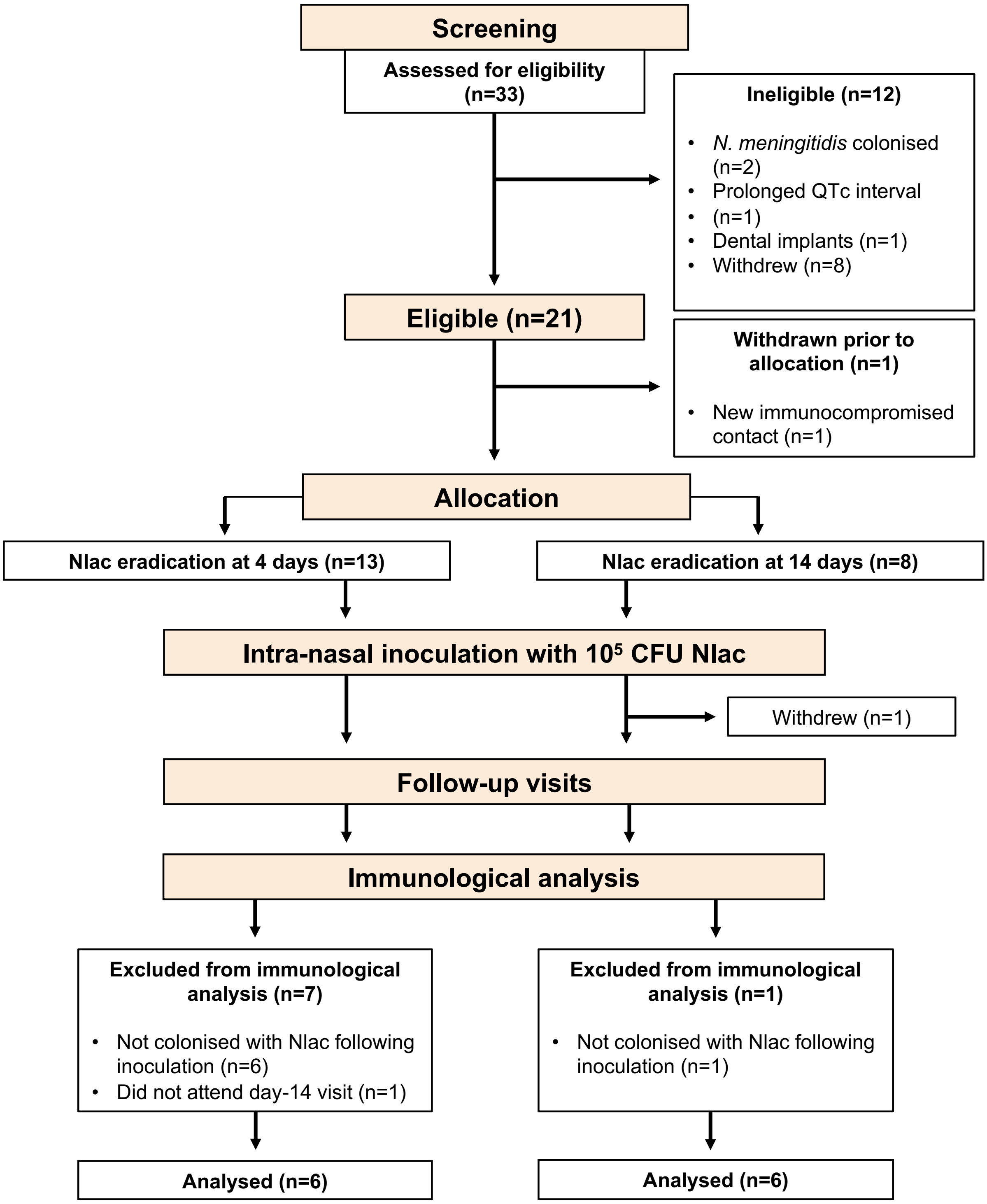

Supplementary Figure 1. Participant flow diagram (Study B). Study flow diagram showing allocation to ciprofloxacin eradication of *N. lactamica* (Nlac) colonisation at 4 days versus 14 days, study completion, and participants included in the immunological analyses. QTc interval determined by assessment of electrocardiogram.

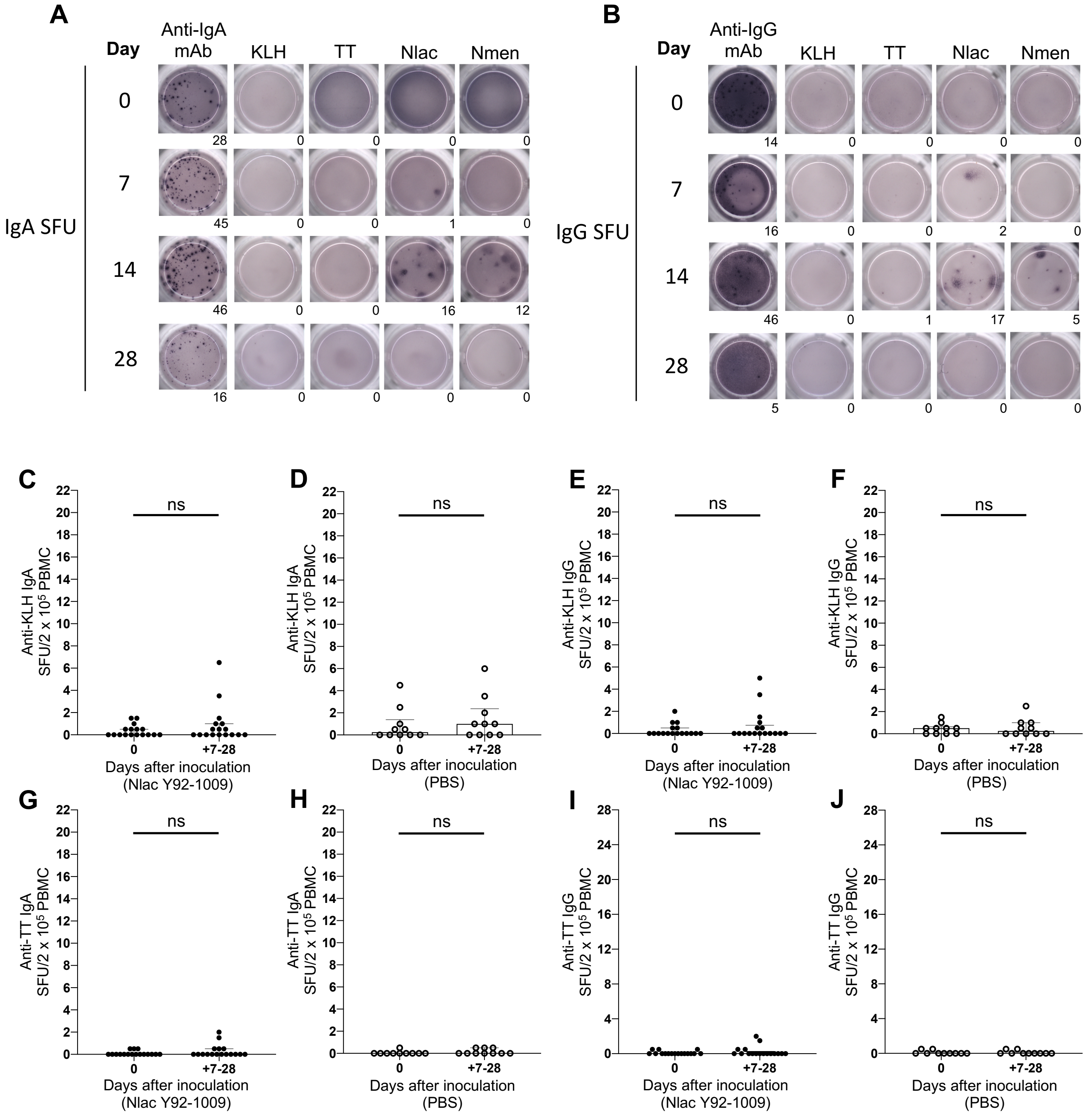

Supplementary Figure 2. Representative B_{PLAS} ELISpot images and pooled KLH-specific and TT-specific B_{PLAS} responses amongst participants. (**A-B**) 2 x 10^5 freshly-isolated PBMC were seeded in duplicate (single well images shown for demonstration purposes) into ELISpot plate wells coated with anti-human IgA mAb (anti-IgA mAb) or anti-human IgG mAb (anti-IgG mAb), keyhole limpet haemocyanin (KLH), tetanus toxoid (TT), Nlac Y92-1009 dOMV (Nlac) and Nmen H44/76-dOMV (Nmen) in 200 µl AIM/V medium. Following an 18-hour incubation, IgA-secreting B_{PLAS} (**A**) and IgG-secreting B_{PLAS} (**B**) were visualised as immunoglobulin spot-forming units (SFU) following incubation with ALK-P-conjugated anti-IgA or anti-IgG pAbs and subsequent incubation with BCIP substrate. IgA and IgG SFU were enumerated using the AID® ELISpot software package, version 3.5, with pre-defined standardised settings (number at bottom right of each image indicates the automated count). (**C-F**) IgA-secreting (**C-D**) and IgG-secreting (**E-F**) B_{PLAS} with specificity to KLH, derived from experimental duplicates, amongst Nlac-colonised (filled circles) and PBS-inoculated (open circles) participants at baseline (day 0) and at the time of 'peak' (day 7, 14 or 28) Nlac-specific B_{PLAS} response. (**G-J**) IgA-secreting (**G-H**) and IgG-secreting (**I-J**) B_{PLAS} with specificity to TT derived from experimental duplicates having adjusted for non-antigen-specific SFU by subtracting the mean SFU enumerated in KLH-coated membranes. For each participant, the highest number of SFU per 2 x 10^5 PBMC is shown (between days 7-28). Columns and error bars represent median and IQR, respectively. ns – not significant, $P < 0.05$, by Wilcoxon matched-pairs signed rank test (n = 17 Nlac-colonised participants, n = 10 PBS-inoculated participants).

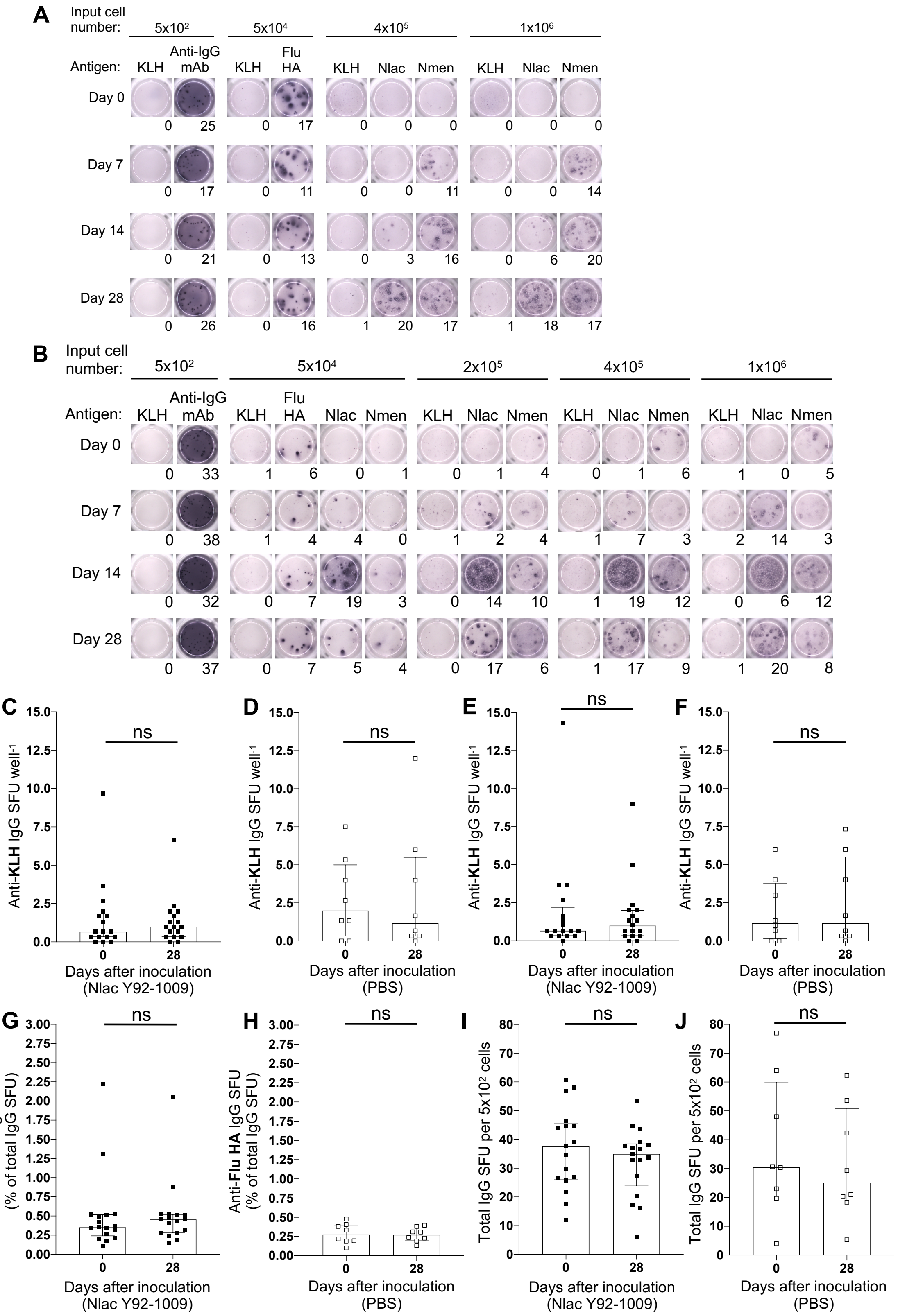

Supplementary Figure 3. Representative IgG B$_{MEM}$ ELISpot images and pooled KLH-specific and Flu HA-specific IgG B$_{MEM}$ responses amongst participants. (**A-B**) PBMC were thawed from LN_2 and polyclonally stimulated at 2 x 10^5 cells per well in 200 µl AIM/V+ medium for 5 days. Cells were harvested and seeded in triplicate (single well images shown for demonstration purposes) into ELISpot plates coated with keyhole limpet haemocyanin (KLH), anti-human IgG mAb (anti-IgG mAb), Influenza antigen reagent 09/174, H1N1 (Flu HA), Nlac Y92-1009-dOMV (Nlac) and Nmen H44/76-dOMV (Nmen) at the numbers outlined in (**A**). Following an 18-hour incubation, IgG-secreting B$_{MEM}$ were visualised as IgG spot-forming

units (SFU) following incubation with ALK-P-conjugated anti-IgG pAb and subsequent incubation with BCIP substrate. IgG SFU were enumerated using the AID® ELISpot software package, version 3.5, with pre-defined standardised settings (number at bottom right of each image indicates the automated count). In cases where PVDF membrane saturation occurred (see day-14 Nlac-coated wells at 4 x 10^5 and 1 x 10^6 input cells - **B**), experiments were repeated with fewer input cells (2 x 10^5 and 5 x 10^4 cells per Nlac/Nmen-coated well) to enable accurate enumeration of IgG SFU (**B**). (**C-F**) Mean IgG SFU enumerated in KLH-coated wells at baseline (day 0) and day 28 amongst Nlac-colonised participants (closed squared) and PBS-inoculated participants (open squares) read at the input cell number where Nlac-specific IgG SFU (**C-D**) and Nmen-specific IgG SFU (**E-F**) were enumerated. (**G-J**) PBMC derived from Nlac-colonised and PBS-inoculated participants were polyclonally stimulated prior to assessment by ELISpot for the presence of IgG-secreting cells with specificity to Flu HA (**G-H**). IgG-secreting cells were visualised as SFU and mean SFU derived from experimental triplicates having adjusted for non-specific SFU by subtracting the mean SFU enumerated in KLH-coated membranes. Antigen-specific IgG-secreting SFU are shown as a percentage of the total number of IgG-secreting SFU (**I-J**) Columns and error bars indicate median and IQR, respectively. Frequencies of IgG-secreting SFU were compared using the Wilcoxon matched-pairs signed rank test (ns – not significant) (*n* = 17 Nlac-colonised participants, *n* = 8 PBS-inoculated participants).

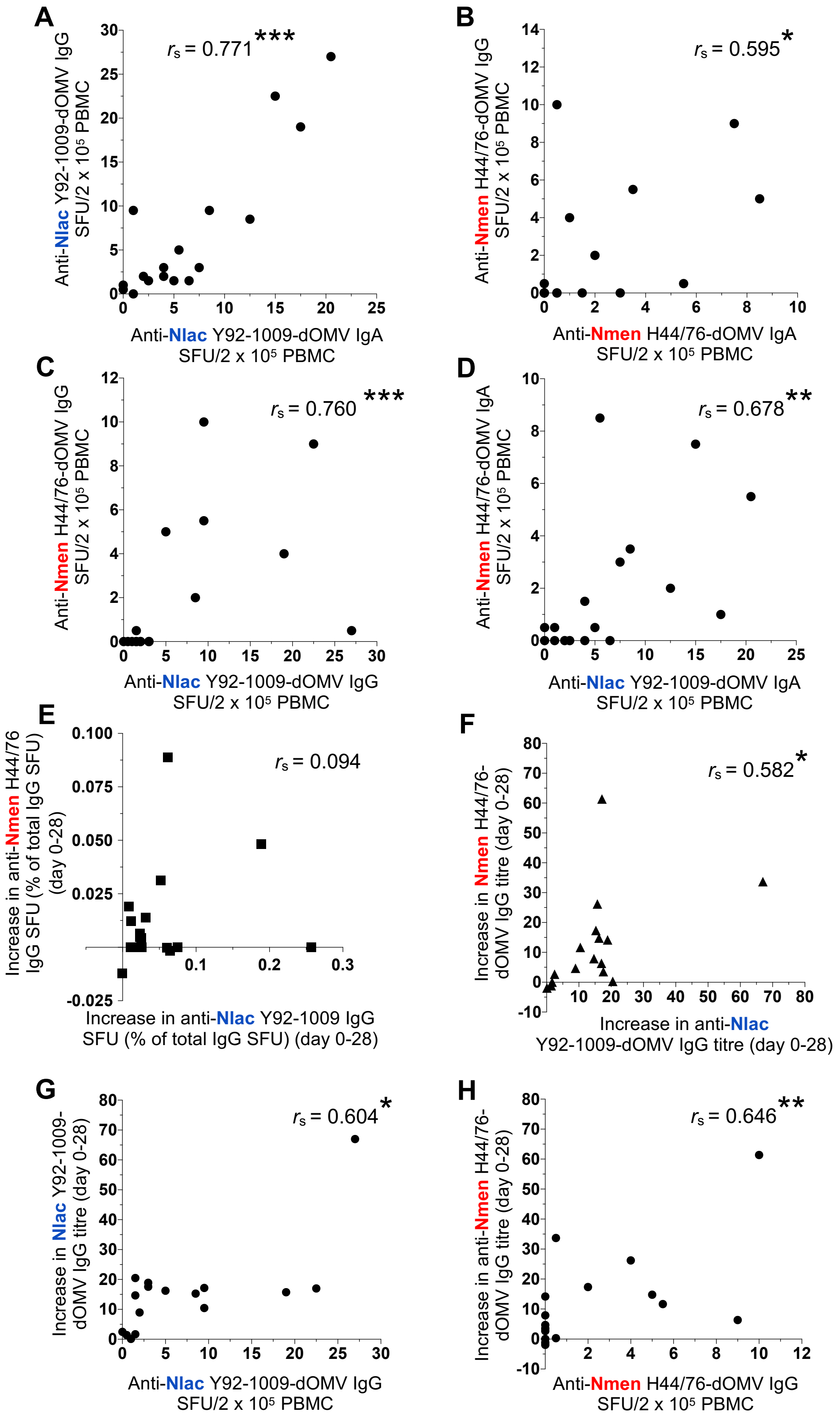

Supplementary Figure 4. Associations between *N. lactamica*- and *N. meningitidis*-specific humoral and B cell responses following *N. lactamica* colonisation. (**A**-**B**) Peak IgA-secreting versus peak IgG-secreting B_{PLAS} frequencies with specificity to Nlac Y92-1009-dOMV (**A**) and Nmen H44/76-dOMV (**B**) following Nlac colonisation (n = 17). (**C**-**D**) Peak Nlac Y92-1009-dOMV-specific versus Nmen H44/76-dOMV-specific IgG-secreting (**C**) and IgA-secreting (**D**) B_{PLAS} frequencies following Nlac colonisation (n = 17). (**E**-**F**) Increase in IgG B_{MEM} frequencies (**E**) (n = 17) and IgG titres (**F**) (n = 16) between baseline (day 0) and day 28 with specificity to Nlac Y92-1009-dOMV and Nmen H44/76-dOMV amongst Nlac-colonised

participants. (**G**-**H**) Increase in IgG titres versus peak IgG-secreting B_{PLAS} frequencies amongst Nlac-colonised participants (*n* = 16) for Nlac Y92-1009-dOMV-specific (**G**) and Nmen H44/76-dOMV-specific (**H**) responses. *P* * ≤ 0.05, ***P* ≤ 0.01, ****P* ≤ 0.00 (Spearman's Rho [r_s]).

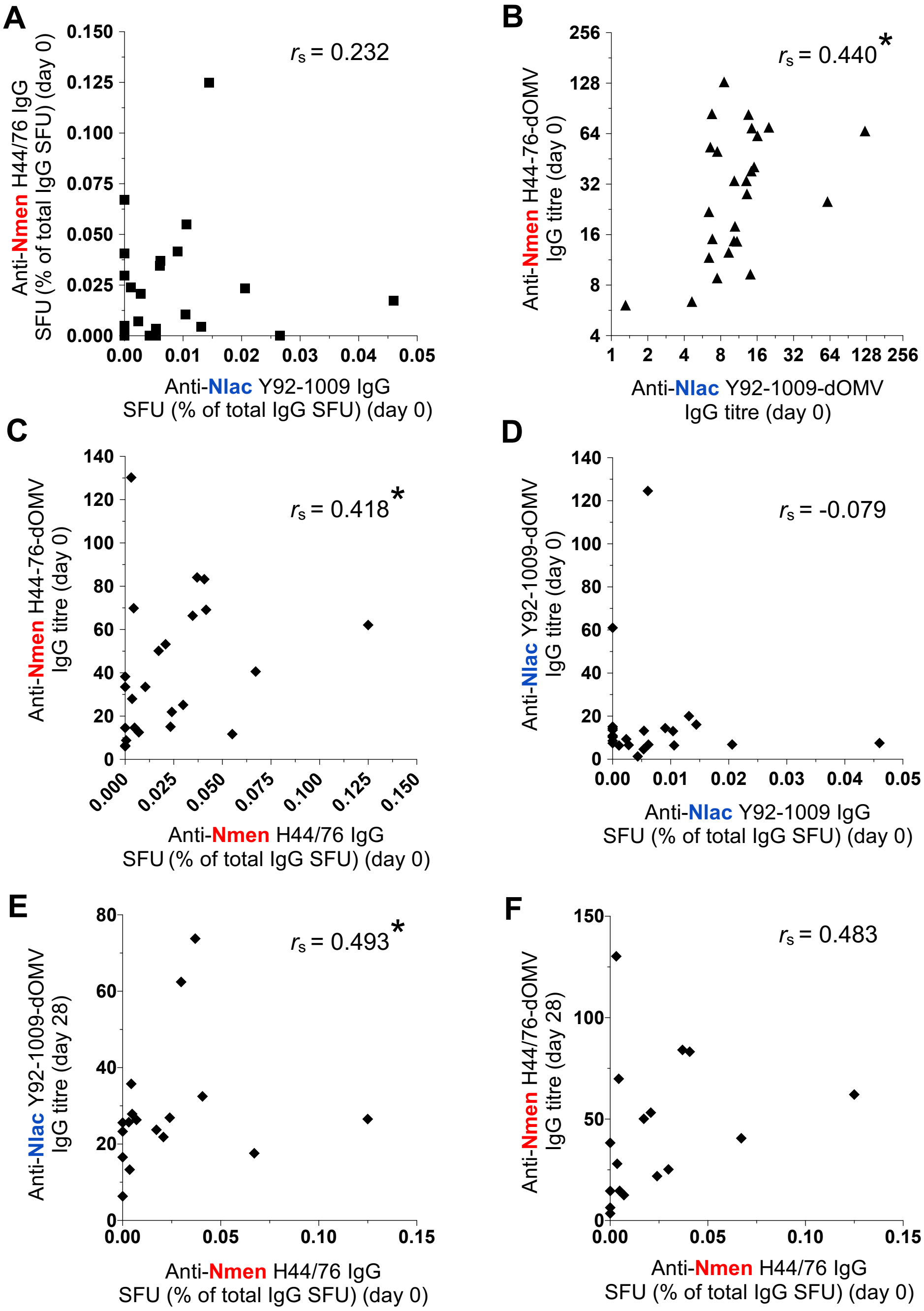

Supplementary Figure 5. Associations between *N. lactamica*- and *N. meningitidis*-specific humoral and B cell responses at baseline. Anti-Nlac Y92-1009-specific and anti-Nmen H44/76-dOMV-specific IgG titres and IgG BMEM frequencies were assessed amongst PBS-inoculated and Nlac-inoculated and colonised participants at baseline (day 0) using ELISA and ELISpot assays, respectively. (**A-B**) Frequency of IgG BMEM ($n = 27$) (**A**) and IgG titres ($n = 26$) (**B**) with specificity to Nlac Y92-1009-dOMV versus Nmen H44/76-dOMV on day 0. (**C-D**) Frequencies of IgG BMEM versus IgG titres on day 0 with specificity to Nmen H44/76-dOMV (**C**) and Nlac Y92-1009-dOMV (**D**) ($n = 26$). (**E-F**) Frequencies of IgG BMEM on day 0 with specificity to Nmen H44/76-dOMV versus day 28 IgG titres with specificity to Nlac Y92-1009-dOMV (**E**) and Nmen H44/76-dOMV (**F**) ($n = 17$). P * ≤ 0.05 (Spearman's Rho [r_s]).

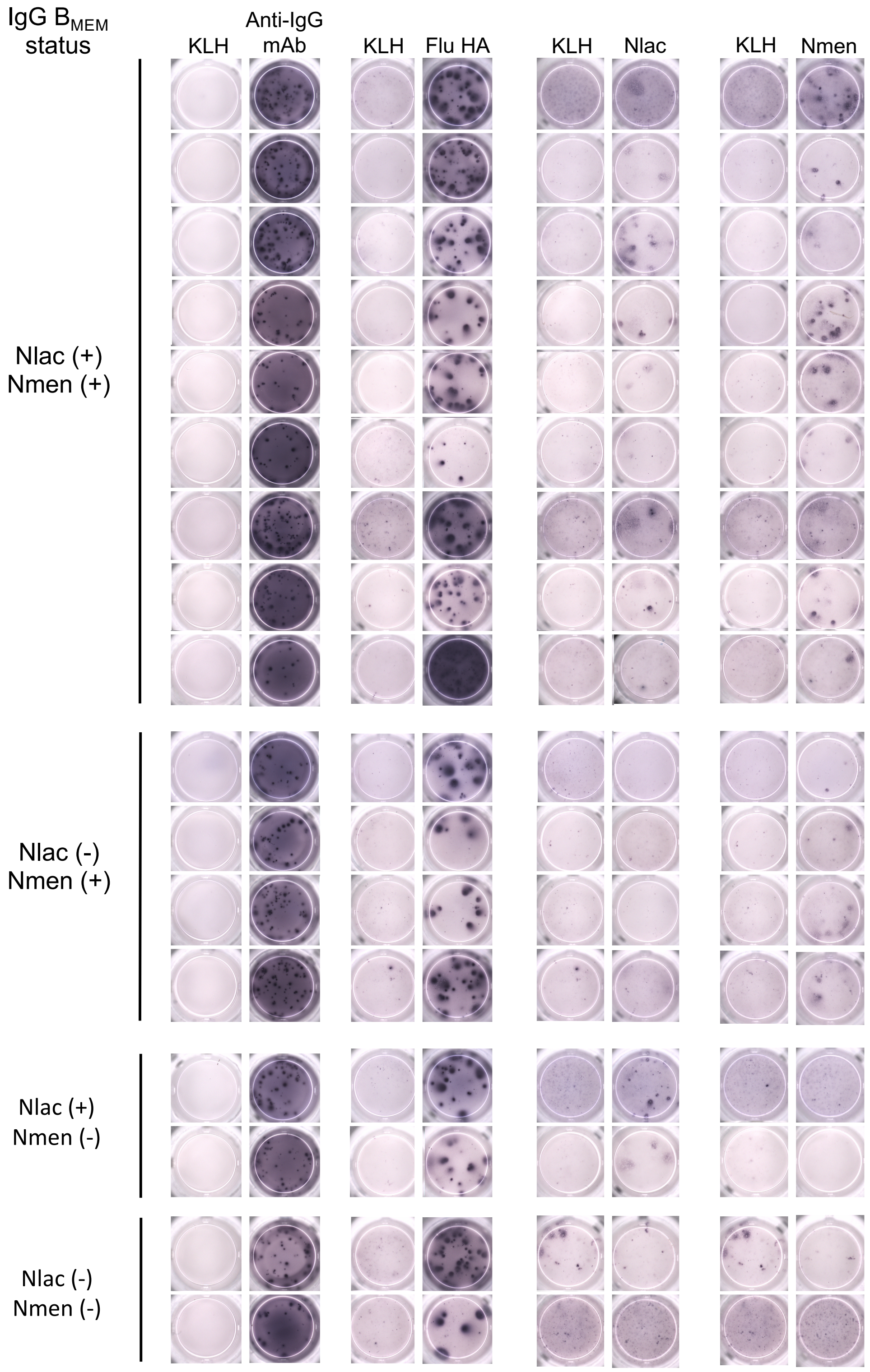

Supplementary Figure 6. IgG B$_{MEM}$ ELISpot data for *N. lactamica*-colonised participants at baseline. Representative IgG B$_{MEM}$ ELISpot well images (single wells shown for demonstration purposes) for *N. lactamica*-colonised participants (n = 17) at baseline showing IgG spot-forming units (SFU) in wells coated with keyhole limpet haemocyanin (KLH), anti-IgG mAb, Influenza antigen reagent 09/174, H1N1 (Flu HA), Nlac Y92-1009-dOMV (Nlac) and Nmen H44/76-dOMV (Nmen). KLH-coated wells shown are matched to the test antigen based on input cell number. Participants were separated into groups based on the presence (+) or absence (-) of IgG B$_{MEM}$ responses specific to Nlac Y92-1009-dOMV and Nmen H44/76-dOMV following automated IgG SFU enumeration using the AID® ELISpot software package, version 3.5, with pre-defined standardised settings, followed by subtraction of the mean SFU count obtained in KLH-coated wells.

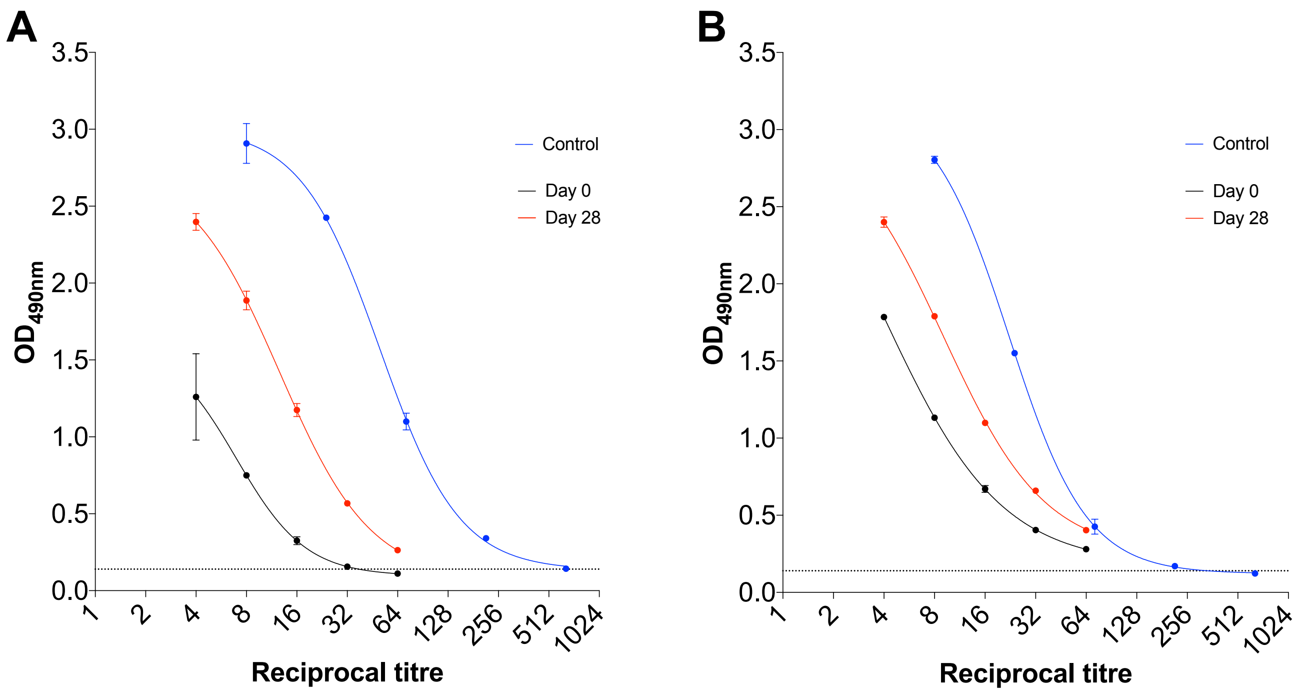

Supplementary Figure 7. Representative plasma IgG ELISA data for a single *N. lactamica*-colonised participant. Plasma samples from a single Nlac-colonised participant and a positive control were serially diluted in duplicate prior to incubation in Nlac Y92-1009-dOMV-coated and Nmen H44/76-dOMV-coated wells of an ELISA plate. Captured IgG was detected as an increase in OD490nm using biotinylated, anti-human IgG mAb, streptavidin-HRP and OPD substrate. Colour change was quantified using a VersaMax plate reader measuring at OD_{490nm}. OD_{490nm} values obtained in Nlac Y92-1009-dOMV-coated wells (**A**) and Nmen H44/76-dOMV-coated wells (**B**) across the dilution series are plotted as mean with SD at baseline (day 0, black circles), day 28 following Nlac inoculation (red circles), and for the positive control (blue circles). IgG titres within day-0 and day-28 plasma were calculated by interpolation from the control dose-response curve. A mean of the acceptable values from all dilutions was then taken as the final value for each test sample with acceptable values defined as those > 3 SDs from the mean OD_{490nm} for BSA-coated wells (= 0.13986) (dotted black line).

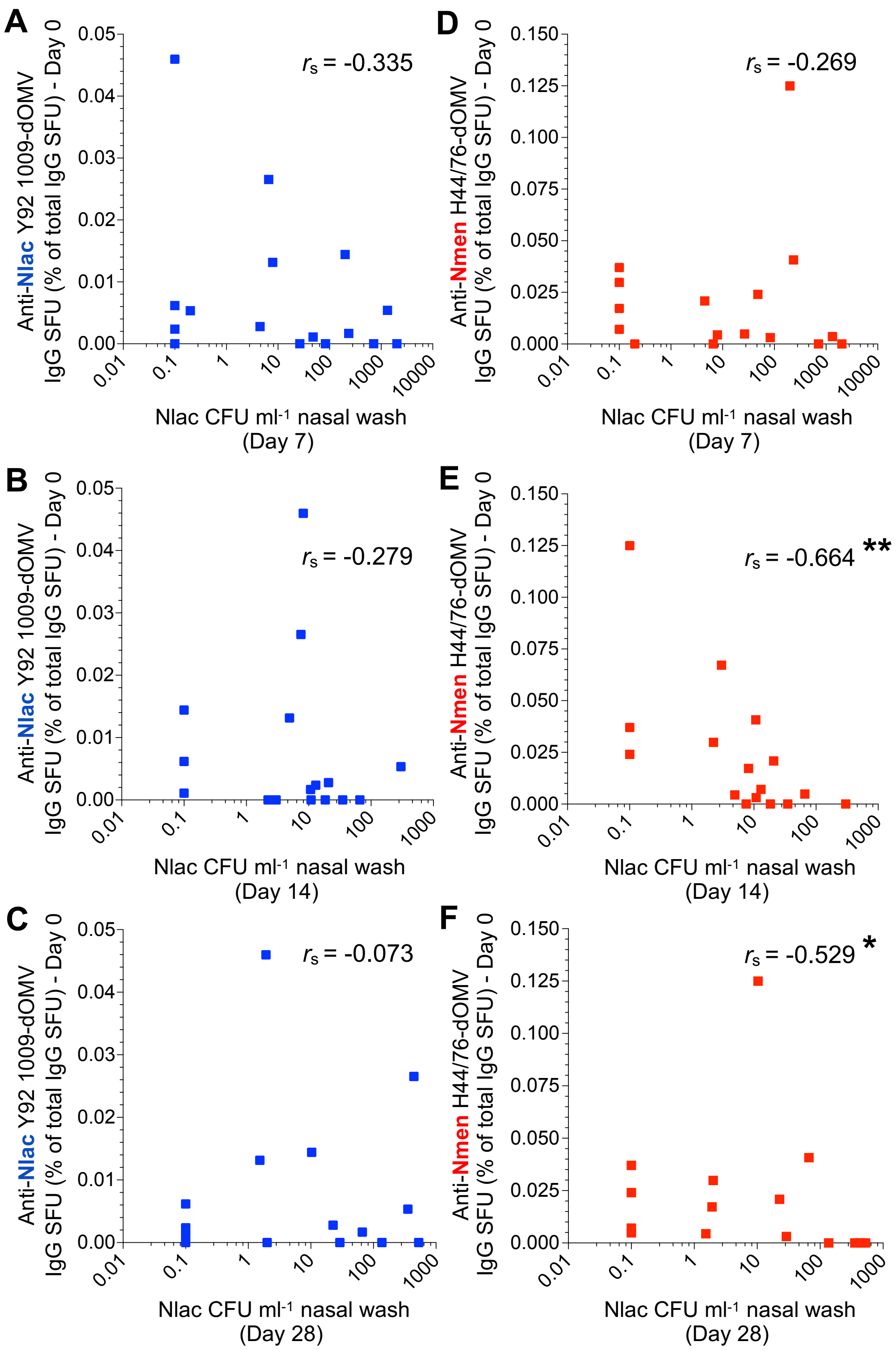

Supplementary Figure 8. Baseline *N. meningitidis*-specific IgG BMEM frequencies inversely correlate with *N. lactamica* colonisation density. Baseline (day 0) IgG BMEM frequencies with specificity to Nlac Y92-1009-dOMV (blue squares) and Nmen H44/76-dOMV (red squares) were determined using the IgG BMEM ELISpot assay, with data displayed as antigen-specific IgG SFU expressed as a percentage of the total number of IgG SFU. IgG BMEM frequencies were plotted against Nlac Y92-1009 CFU ml^{-1} data derived from the nasal wash specimens of Nlac-colonised participants at 7 days (**A, D**), 14 days (**B, E**) and 28 days (**C, F**) post-inoculation. Nlac CFU ml^{-1} values of 0 were set to 0.01 to allow visualisation of all data points in log_{10} format. Correlations were assessed with Spearman's Rho (r_s) ($*P \leq 0.05$, $**P \leq 0.01$).

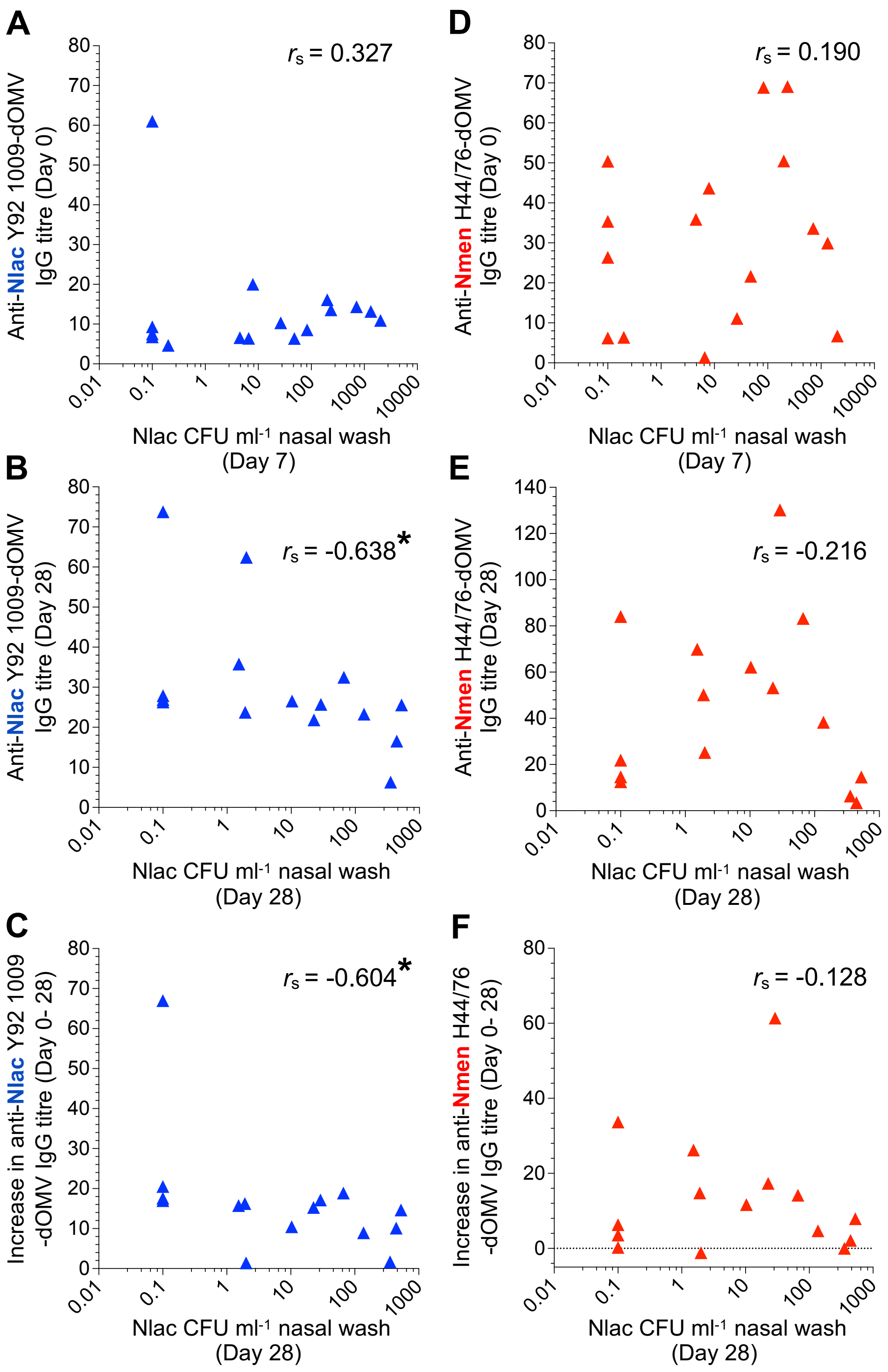

Supplementary Figure 9. *N. lactamica*-specific IgG titres inversely correlate with *N. lactamica* colonisation density. IgG titres with specificity to Nlac Y92-1009-dOMV (blue triangles) and Nmen H44/76-dOMV (red triangles) were determined amongst Nlac-colonised participants at day 0 and day 28 following Nlac inoculation using an ELISA and plotted against Nlac Y92-1009 CFU ml^{-1} in nasal wash at day 7 (**A**, **D**) and day 28 (**B**, **E**). Nlac Y92-1009 CFU ml^{-1} in nasal wash at day 28 plotted against the increase in IgG titres between day 0-28 with specificity to Nlac Y92-1009-dOMV (**C**) and Nmen H44/76-dOMV (**F**) amongst Nlac-colonised participants. Nlac CFU ml^{-1} values of 0 were set to 0.01 to allow visualisation of all data points in log_{10} format. Correlations were assessed with Spearman's Rho (r_s) (*$P \leq 0.05$).

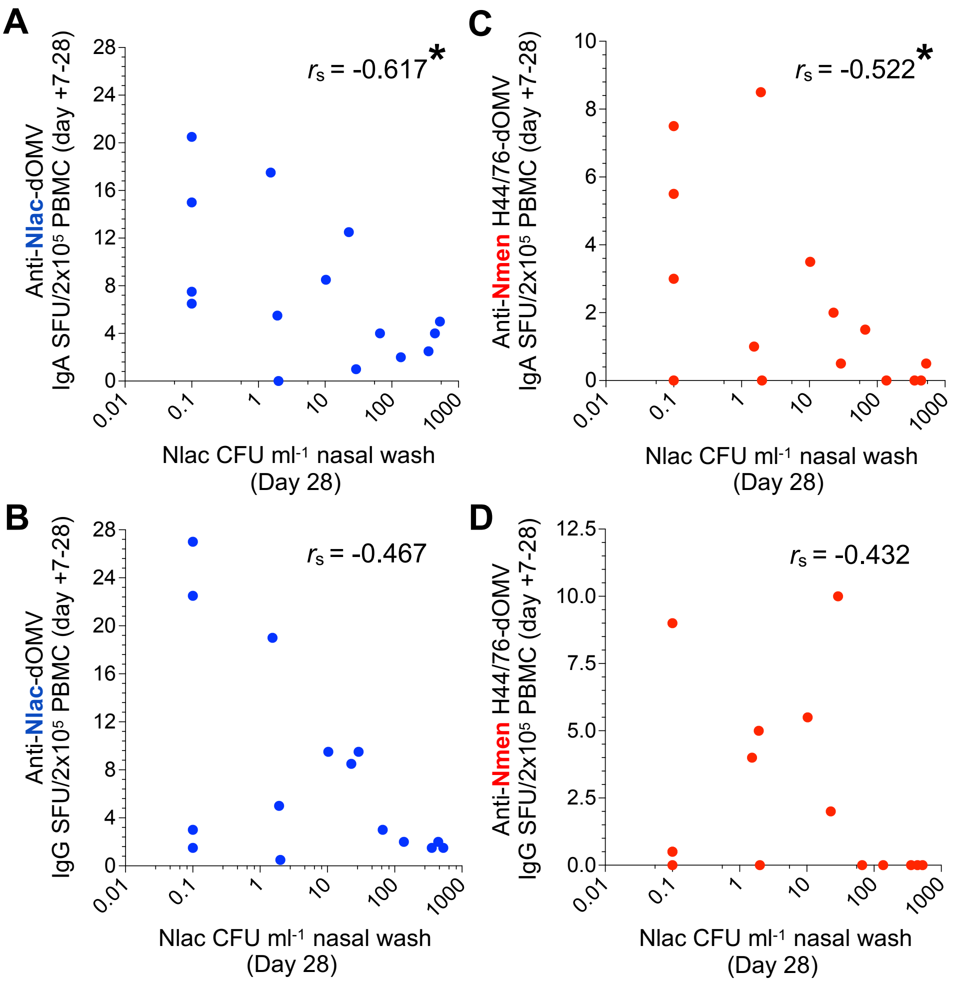

Supplementary Figure 10. ***N. lactamica*-specific IgA-secreting BPLAS frequencies inversely correlate with *N. lactamica* colonisation density.** Peak IgA-secreting and IgG-secreting BPLAS frequencies (day +7-28) with specificity to Nlac Y92-1009 dOMV (blue circles) (**A**-**B**) and Nmen H44/76 dOMV (red circles) (**C**-**D**) were determined amongst Nlac-colonised participants using BPLAS ELISpot assays. Correlations between BPLAS frequencies and Nlac Y92-1009 CFU ml^{-1} data derived from nasal wash on day 28 were assessed with Spearman's Rho (r_s) (*$P \leq 0.05$). Nlac CFU ml^{-1} values of 0 were set to 0.01 to allow visualisation of all data points in log_{10} format.

**Study A**	Screening	Inoculation	Follow-up		
Timeline (days, +/- window)	-28 to -7	0	+7 (+/-3)	+14 (+/-3)	+28 (+/-5)
Informed consent	+				
Medical history	+				
Vital signs		+			
Physical examination	(+)	(+)	(+)	(+)	(+)
Pregnancy test (females only)	+	(+)	(+)	(+)	(+)
Review eligibility		+			
Intra-nasal inoculation with *N. lactamica* or PBS		+			
Nasal wash (Neisseria spp. culture)	+		+	+	+
Oropharyngeal throat swab (Neisseria spp. culture)	+	+	+	+	+
Baseline Haemoglobin (blood)	+				
Venesection		+	+	+	+

Supplementary Table 1. Study A clinical procedures. + Always performed, (+) performed if clinically-indicated.

**Study B – Nlac eradication at 4 days**	Screening	Inoculation	Follow-up			
Timeline (days, +/- window)	≤ 90	0	4	5	14 (+/- 2)	32 (+/- 2)
Informed consent	+					
Medical history	+					
Vital signs	+	+	(+)	(+)	(+)	(+)
Physical examination	+	(+)	(+)	(+)	(+)	(+)
Pregnancy test (females only)	+	+	+			
Electrocardiogram	+	+[a]				
Review eligibility		+				
Intra-nasal inoculation with *N. lactamica*		+				
Eradication (ciprofloxacin, 500mg)			+			
Nasal wash (Neisseria spp. culture)	+				+	+
Oropharyngeal throat swab (Neisseria spp. culture)	+	+	+	+	+	+
Venesection		+			+	+

**Study B - Nlac eradication at 14 days**	Screening	Inoculation	Follow-up						
Timeline (days, +/- window)	≤ 90	0	4	5	7 (+/- 2)	14 (+/- 2)	15	24 (+/- 2)	42 (+/- 2)
Informed consent	+								
Medical history	+								
Vital signs	+	+	(+)	(+)	(+)	(+)	(+)	(+)	(+)
Physical examination	+	(+)	(+)	(+)	(+)	(+)	(+)	(+)	(+)
Pregnancy test (females only)	+	+				+			
Electrocardiogram	+	+[a]							
Review eligibility		+							
Intra-nasal inoculation with N. lactamica or PBS		+							
Eradication (ciprofloxacin, 500mg)						+			
Nasal wash (Neisseria spp. culture)	+					+			+
Oropharyngeal throat swab (Neisseria spp. culture)	+	+	+	+	+	+	+	+	+
Venesection		+				+			+

Supplementary Table 2. Study B clinical procedures. + Always performed, (+) performed if clinically-indicated, [a] performed if not available from screening visit.

**Inclusion criteria**	
•	Healthy adults aged 18 to 45 years inclusive on the day of enrolment.
•	Fully conversant in the English language.
•	Able and willing (in the investigator's opinion) to comply with all study requirements.
•	Written informed consent to participate in the study.
•	For females only, willingness to practice continuous effective contraception (see below) during the study and negative pregnancy test at visit 1 (screening).
•	Willingness to take an antibiotic regimen after inoculation according to the study protocol[†]
**Exclusion criteria**	
•	Active smokers
•	*N. meningitidis* or *N. lactamica* detected following culture of throat swab or nasal wash taken before the challenge.
•	Individuals who have a current infection at the time of inoculation.
•	Individuals who have been involved in other clinical studies/trials involving receipt of an investigational product over the last 12 weeks or if there is planned use of an investigational product during the study period.
•	Individuals who have previously been involved in clinical studies/trials investigating meningococcal vaccines or experimental challenge with *N. lactamica.*
•	Use of oral or intravenous antibiotics within the period 30 days prior to the challenge.
•	Any confirmed or suspected immunosuppressive or immunocompromised state, including HIV infection, asplenia, history of recurrent severe infections or use (more than 14 days) of immunosuppressant medication within the past 6 months (topical/inhaled steroids are allowed).
•	Use of immunoglobulins or blood products within 3 months prior to enrolment.
•	History of blood donation within the past 12 weeks for male volunteers, or 16 weeks for female volunteers[‡].
•	Allergy to yeast extract.
•	Contraindications to the use of ciprofloxacin, specifically a history of epilepsy, prolonged QT interval, hypersensitivity to quinolones or a history of tendon disorders related to quinolone use[†].
•	Any other significant disease, disorder, or finding which may significantly increase the risk to the volunteer because of participation in the study, affect the ability of the volunteer to participate in the study, or impair interpretation of the study data, for example recent surgery to the nasopharynx.
•	Occupational, household or intimate contact with immunosuppressed persons.
•	Pregnancy or lactation.

Supplementary Table 3. Inclusion and exclusion criteria for the *N. lactamica* (Y92-1009) CHIMEs. [‡] Criteria specific to study A. [†] Criteria specific to Study B.

Participant	Assignment	Adverse event	Symptom(s) start date following inoculation visit (days)	Severeity grade (1-3)	Duration (days)	Relatedness to intervention (*i.e.*, intra-nasal inoculation)
**2**	Nlac	Sore throat and cough. Headache for one day only.	15	2	5	Unlikely related
**5**	Nlac	Cough, runny nose.	19	2	3	Unlikely related
**6**	Nlac	Nasal stuffiness and runny nose consistent with hayfever symptoms prior to and on the day of intra-nasal inoculation (participant known to have hayfever).	Pre-inoculation	1	1	No relationship
**6**	Nlac	Sore throat, headache, nasal stuffiness and runny nose.	12	1	2	Unlikely related
**8**	Nlac	Microbiologically-confirmed urinary tract infection, possibly ascending.	6	3	6	No relationship
**17**	Nlac	Intermittent headache throughout study duration (known chronic intermittent headache) including prior to and on the day of intra-nasal inoculation.	Pre-inoculation	1	Intermittent	No relationship
**17**	Nlac	Runny nose.	28	1	1	Unlikely related
**21**	Nlac	Sore throat, headache, nasal stuffiness, runny nose.	6	2	14	Unlikely related
**23**	Nlac	Runny nose, cough, nasal stufiness and intermittent headache.	23	1	1	Unlikely related
**24**	Nlac	Nasal stuffiness and runny nose with intermittent headache prior to and on the day of intra-nasal inoculation.	Pre-inoculation	2	14	No relationship
**26**	Nlac	Nasal stuffiness only.	14	1	14	Unlikely related
**27**	Nlac	Participant snorted during inoculation, causing some nasal secretion containing inoculum to run into participant's eye. Eye washed with water, no immediate or longer-term conseqeunces noted.	Pre-inoculation	1	NA	Related
**27**	Nlac	Sore throat only.	14	1	1	Unlikely related
**29**	Nlac	Nasal stuffiness only prior to and on the day of intra-nasal inoculation.	Pre-inoculation	1	3	No relationship
**9**	PBS	Nasal stuffiness and runny nose.	15	1	4	Unlikely related
**14**	PBS	Runny nose and nasal stuffiness.	7	1	7	Unlikley related
**16**	PBS	Pre-syncopal episode following venepuncture and prior to intra-nasal inoculution. Recovered wihin 4-5 minutes and proceeded to inoculation.	Pre-inoculation	1	NA	No relationship (*i.e.*, related to venepuncture only)
**16**	PBS	Runny nose only.	28	1	1	Unlikely related
**22**	PBS	Runny nose, nasal stuffiness, headache and cough.	25	2	3	Unlikely related
**28**	PBS	Runny nose and nasal stuffienss prior to and on day of intra-nasal inoculation.	Pre-inoculation	1	1	No relationship
**31**	PBS	Sore throat, congested, runny nose on day of and prior to intra-nasal inoculation.	Pre-inoculation	2	7	Unlikely related

Supplementary Table 4. Study A adverse events. Timing, duration and severity of adverse events reported amongst Study A participants assigned to intervention (Nlac) or control (PBS). Severity of adverse events and their relatedness to the intervention were assigned in line with procedures set out in the Clinical Study Protocol.

Participant	Assignment	Adverse event	Symptom(s) start date following inoculation visit (days)	Severity grade (1-3)	Duration (days)	Relatedness to intervention (*i.e.*, intra-nasal inoculation)
**5**	Group 1	Foot pain requiring ibuprofen, likley due to running. Started prior to day of inoculation.	Pre-inoculation	2	14	No relationship
**6**	Group 1	Nasal congestion, contact with family members with viral upper respiratory tract infections. Took a dose of an antihistamine.	1	2	1	Possibly related
**10**	Group 1	Slight sore throat following throat swabs. Resolved rapidly, no treatment necessary.	4	1	1	No relationship (*i.e.*, related to throat swab precedure only)
**10**	Group 1	Coryzal illness, cough, systemically well.	16	1	27	Unlikely related
**13**	Group 1	Self-isolated with possible COVID-19, took paracetamol for 2 days and fuly recovered.	12	2	2	Unlikley related
**14**	Group 2	Felt run down, general malaise. Took 1 dose of ibuprofen.	23	2	1	No relationship
**15**	Group 2	Dysuria/cystitis requiring paracetamol for 2 days.	41	2	2	No relationship
**16**	Group 2	Cyst present behind ear prior to study involvement. Required drainage prior to study enrollment and healed over course of study. No antibiotics taken.	Pre-inoculation	2	14	No relationship
**17**	Group 2	Lethargy prior to and on day of intra-nasal inoculation (in context of very hot weather).	Pre-inoculation	1	15	Unlikely related
**17**	Group 2	Erythematous pharynx and cervical lymphadenopathy prior to and on day of intra-nasal inoculation.	Pre-inoculation	1	7	Unlikely related
**18**	Group 2	Headache, not limiting activity but took 1 dose paracetamol (prior to day 14 ciprofloxacin).	14	2	1	Unlikely related
**19**	Group 2	Vomiting. Visited GP and received ibuprofen, dioralyte and antiemetics (intra-muscular and orally) (prior to day 14 ciprofloxacin).	14	3	1	Unlikely related
**19**	Group 2	Diarrhoea (prior to day 14 ciprofloxacin).	14	3	1	Unlikely related
**20**	Group 2	Sore throat, resolved after a drink.	15	1	1	Unlikely related
**20**	Group 2	Nasal congestion, no fever, resolved spontaneously. Had lemsip.	17	2	7	Unlikely related

Supplementary Table 5. Study B adverse events. Timing, duration and severity of adverse events reported amongst Study B participants assigned to Nlac colonisation for four days (Group 1) or 14 days (Group 2). Severity of adverse events and their relatedness to the intervention were assigned in line with procedures set out in the Clinical Study Protocol.

Supplementary Appendix 1. Sample size and power calculation for Study B.

In a previous Nlac challenge study a significant rise in Nlac-specific IgG titre in serum was observed over 2 weeks of infection.[11] Total Nlac-specific serum IgG exhibited a standard deviation on a log-10 scale of 0.26. In the current study, using the standard deviation of 0.26, we calculated that we would be able to confirm a 4-fold rise in Nlac-specific IgG titre with 10 carriers of Nlac with 90 % power using analysis of variance.

The sample size and power calculation for Study B was derived prior to serological data derived from Study A being fully analysed; *a priori* an ordinal readout for the IgG ELISA of Study B had been planned (*i.e.* of 1:2, 1:4, 1:8 etc). At this time, it was considered that a 4-fold increase in end-point titre, for example 1:2 to 1:8, was the most appropriate endpoint, taking into account assay reliability (there were concerns that if a 2-fold change was utilised as the endpoint with this ordinal-type assay, variability in assay performance could result in difficulties with interpretation as the proportion of participants with >2-fold change due to assay variability alone may be high). It has become apparent that a four-fold rise in Nlac-specific IgG titre was an inappropriate endpoint. There are two main reasons for this:

(1) In the majority of Nlac-colonised participants, the increase in Nlac-dOMV-specific IgG titre is <4-fold. For example, in Study A, Nlac-dOMV IgG titres increased by >4-fold in only n = 2/16 Nlac-colonised volunteers between day 0 and day 28 (Median fold-change 2.4-fold, IQR 1.4-3.1).

(2) Since this initial sample size and power calculation, and subsequent to cessation of Study B, we have significantly improved the IgG ELISA - note the low variability in Nlac-dOMV-specific IgG titres observed amongst PBS-inoculated participants in Study A (Median fold change in Nlac-dOMV-specific IgG between day 0 and day 8 = 1.021 [IQR 0.97-1.156]). In addition, we have changed our approach so that an interpolated titre, in the form of continuous data, is now calculated using standard positive control serum (see methods section for further details). Generation of continuous data from this highly accurate assay meant that we no longer had to use the blunt ordinal readout to separate control and intervention groups into 'responders' vs. 'non-responders' based on presence or absence of a 4-fold rise in Nlac-dOMV-specific IgG titre. The main advantage of this is that it provides more granularity to the change in Nlac-dOMV-specific IgG titres that has occurred.

Taking both points above into account, the authors felt that it was no longer appropriate to use the end point of 'at least a 4-fold rise in Nlac-dOMV-specific IgG titre' to categorise Study B participants into 'responder' and 'non-responder' groups. Instead, the authors agreed to show the IgG titres, as calculated, in continuous data format, with an appropriate statistical test for this data type.
